# Characterization of hepatocellular carcinoma patient-derived organoids from patients receiving transarterial chemoembolization as a model for preclinical drug response assessment

**DOI:** 10.1038/s41598-026-65418-3

**Published:** 2026-08-04

**Authors:** Jaafar Khaled, Sofi Sennefelt Nyman, David Dahlgren, Fredrik Rorsman, Hans Lennernäs, Charlotte Ebeling Barbier, Femke Heindryckx

**Affiliations:** 1 Department of Medical Cell Biology, Uppsala University, Husargatan 3, 75123 Uppsala, Sweden; 2 Department of Surgical Sciences, Section of Radiology, Uppsala University, Uppsala, Sweden; 3 Translational Drug Development and Discovery, Department of Pharmaceutical Biosciences, Uppsala University, Uppsala, USA; 4 Department of Medical Sciences, Uppsala University, Uppsala, Sweden

**Keywords:** Hepatocellular carcinoma, Transarterial chemoembolization, Patient-derived organoids, Idarubicin, Drug response, Cancer, Oncology

## Abstract

Hepatocellular carcinoma (HCC) is the most frequent type of primary liver cancer and is often diagnosed at an intermediate stage in patients with chronic liver disease. Transarterial chemoembolization (TACE) is the first-line treatment in this setting. However, drug response remains variable and optimal chemotherapeutic selection unresolved. Idarubicin (IDA) has previously demonstrated antitumor activity comparable to doxorubicin (DOX), with improved emulsion stability and membrane permeability. 3D patient-derived organoids (PDOs) have also recently emerged as physiologically relevant systems that recapitulate tumor characteristics and inter-patient heterogeneity more accurately than conventional 2D cell cultures. In this study, we characterized HCC PDOs and investigated their utility as an ex vivo model to assess therapeutic responses. We generated organoids from tumor and non-tumor liver biopsies collected from HCC patients prior to TACE-treatment. PDOs partially preserved tumor architecture and phenotypic features. IDA exposure resulted in a concentration-dependent reduction in organoid growth, with marked inter-patient variability in drug response. Comparative analyses showed that IDA was more potent than DOX, with lower IC₅₀ values across all patient-derived samples. IDA also induced transcriptional changes associated with cellular stress, inflammation, and proliferation pathways. However, ex vivo drug‑sensitivity testing in PDOs did not correlate with clinical response to TACE. These findings confirm the feasibility of generating HCC PDOs and using them to evaluate differential sensitivity to TACE‑associated chemotherapeutic agents ex vivo, although their ability to predict clinical efficacy remains unproven. While IDA exhibited greater cytotoxic activity than DOX in organoid cultures, this observation was based on in vitro sensitivity assays and does not establish clinical superiority. Furthermore, the absence of correlation between PDO drug sensitivity and clinical TACE response indicates that PDO-based prediction of treatment outcome remains challenging in this setting.

**Supplementary Information:**

The online version contains supplementary material available at 10.1038/s41598-026-65418-3.

## Introduction

Hepatocellular carcinoma (HCC) is the most frequent primary liver malignancy, comprising nearly 90% of cases worldwide. It represents a major health challenge and a leading cause of cancer-related deaths^1,2^. HCC typically occurs in association with underlying chronic liver damage, causing inflammation, reversible fibrosis, and ultimately non-reversible cirrhosis^3,4^. Obesity, Metabolic Dysfunction-Associated Steatotic Liver Disease (MASLD) and alcoholic liver diseases are among the etiological causes of HCC that are increasing rapidly in incidence, particularly in Western countries^2^. Concurrently, hepatitis B and C virus infections, as well as Metabolic Dysfunction-Associated Steatohepatitis (MASH) are also among the leading HCC risk factors^1,5^. Limitations in the sensitivity of current diagnostic methods for detecting early HCC in cirrhotic livers often result in diagnosis at an intermediate stage^6,7^. Accordingly, the standard recommended therapy for intermediate HCC is transarterial chemoembolization (TACE). TACE is a locoregional treatment in which a chemotherapeutic agent, typically emulsified in an oily carrier such as Lipiodol^®^, is directly delivered to the tumor via arterial injection, followed by embolization of the tumor-feeding arteries. This combined approach induces tumor necrosis by both the cytotoxic action of the drug and ischemia from blood flow interruption, while also prolonging local drug retention and exposure^6^. Although systemic treatments have been widely investigated, patient survival remains low, undermining the efficacy of existing HCC management^8^. A clinical advantage with locoregional cytotoxic treatments, such as TACE, is that systemic side-effects such as intestinal mucositis and diarrhea might be avoided or reduced^9,10^. However, there is still a fundamental need to develop novel therapeutic approaches for HCC and to improve TACE-efficacy.

Several cytotoxic drugs are currently available for TACE, such as cisplatin, epirubicin, doxorubicin (DOX) and idarubicin (IDA), with DOX being generally the most prevalent chemotherapeutic choice^11^. Yet, no clear rationale has been put forward to account for the ubiquity of DOX as a preferred treatment option^12^. Studies have suggested that IDA and DOX are equally potent and safe when used in TACE^13,14^. IDA has been found to exhibit higher efficacy in vitro when compared to other chemotherapeutic agents, including DOX^15^, although recent in vitro findings reported equal effects on tumor area in DEN-induced mice^16^. Furthermore, IDA, (Log *P* = 1.69), has been shown to be more stable in emulsion than DOX, as well as providing a higher cell membrane permeability^17,18^.

Preclinical assessment of anticancer drugs has been traditionally performed using human liver cancer cell lines grown in two-dimensional (2D) cell culture conditions. Nevertheless, there are significant limitations inherent to this cell culture model, as it is unable to reproduce essential morphological and phenotypical attributes associated with malignant tissues, such as tumor three-dimensionality, tumor cell renewal and intercellular interactions^19,20^. Several studies have focused on the development and implementation of 3D culture models that could potentially mimic the biological conditions of human cancer cells and be used to anticipate therapeutic outcomes in patients^19,21–24^. In recent years, there has been a particular interest in generating ex vivo patient-derived organoid (PDO) systems^20,23^. Such advanced 3D ex vivo models have been found to preserve tumor-specific physiological, genetical and transcriptional characteristics, including cell heterogeneity, cell-to-cell interplay, cell invasion and cell differentiation^19,23,24^.

The purpose of this study was therefore to evaluate the potential of human patient-derived organoids (PDOs) as an ex vivo preclinical system to model therapeutic response in HCC. The morphological and phenotypic features of human derived PDOs were characterized to assess their ability to recapitulate tumor architecture and cellular heterogeneity. Furthermore, the response to IDA was investigated, alongside its benefits as a possible alternative and more effective chemotherapeutic option in comparison to DOX.

## Methods

### TACTida clinical trial

This study is part of a larger clinical trial involving HCC patients undergoing TACE treatment with an infusion of idarubicin (TACTida). The TACTida study is an open-label, single center, non-randomized two-step active-treatment clinical trial. The study protocol has been previously documented by Nyman et al.^25^. The trial is registered in the European Union Clinical Trials Register under EUCTR2021-001257-31-SE (registration date: 02 July 2021) and approved by the Swedish Ethical Review Authority (Dnr. 2021 − 01928) and the Medical Products Agency, Uppsala, Sweden. The study was conducted in accordance with the Declaration of Helsinki. Patients were recruited and biopsies collected at the Uppsala University Hospital, Sweden, between February 2022 and November 2024, in accordance with the inclusion and the exclusion criteria stated in the study protocol^25^. Study completion was achieved in June 2025. Following written and oral information regarding the study, an informed consent form was signed by all participants. No patients or members of the public were involved in the design or conduct of the study. TACE was conducted by experienced interventional radiologists at the Uppsala University Hospital following local standard practice.

### Human tissues and biopsy procedure

**Fig. 1:**
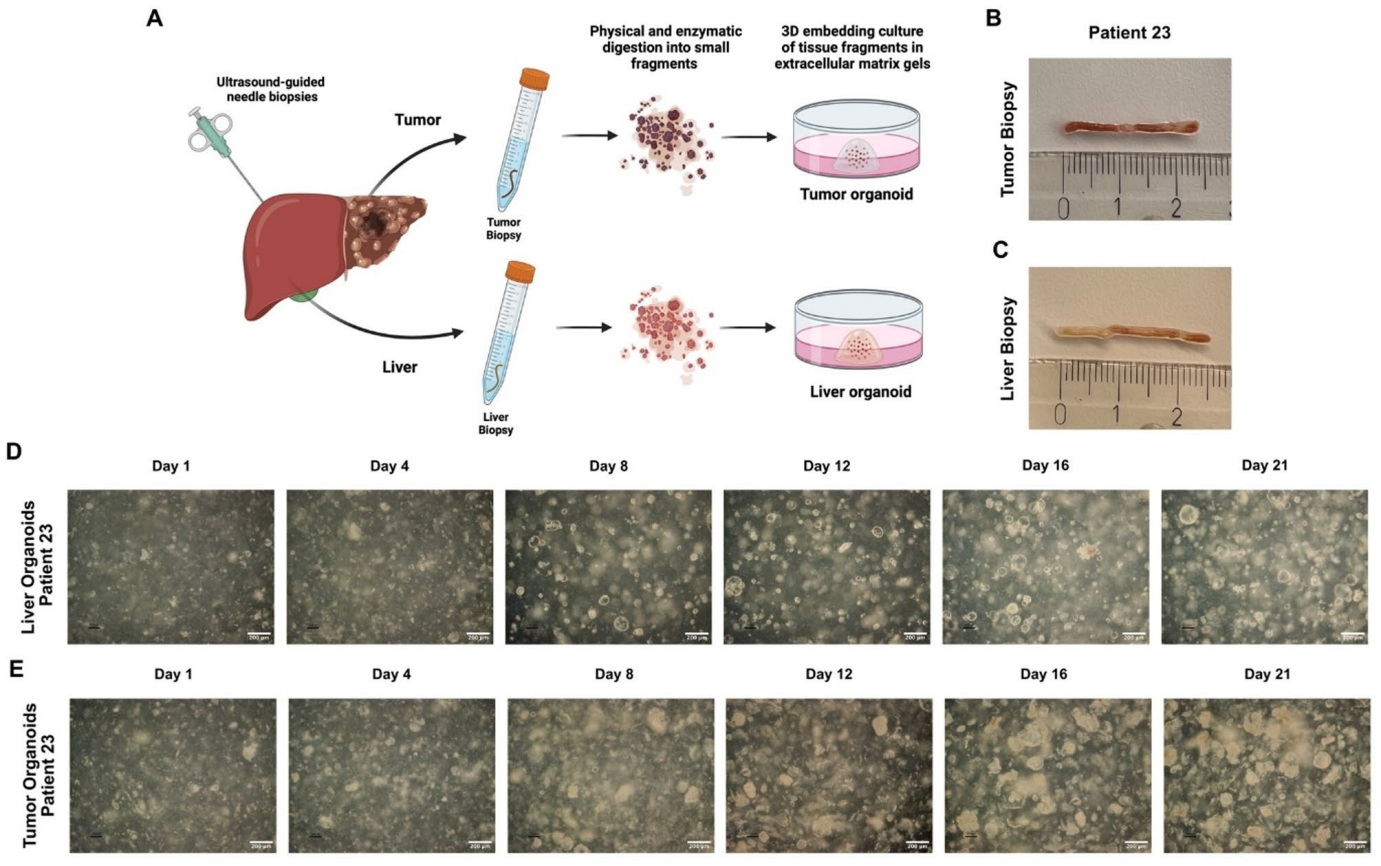
Establishment of tumoral and non-tumoral organoid cultures from needle biopsies of HCC Patients. **(A)** Schematic workflow of organoid generation from needle biopsies. Representative biopsy images of tumor **(B)** and liver tissues **(C)** used for organoid generation. Representative bright-field images of non-tumor **(D)** and paired tumor liver tissue **(E)** organoids from patient 23 during culture. Tumor organoids form compact spheroids, whereas liver organoids from non-tumor liver tissue grow as cystic structures. Organoids were imaged regularly during the initiation period through Day 21 (end of initiation period). Scale bar: 200 μm.

The present research was conducted in collaboration with Uppsala University Hospital, Sweden, which provided the human biopsies. Tumoral tissue and non-tumoral liver parenchymal samples were acquired from biopsies performed on patients diagnosed with HCC enrolled in the TACTida trial. As described by Nyman et al.^25^, 1.6 mm diameter ultrasound-guided needle liver biopsies were obtained under local anesthesia, following each PET/MRI scan. Imaging was performed on each hepatic lobe to retrieve a total of four liver biopsies. Two biopsies were harvested from a viable tumor, followed by two other biopsies collected from non-tumorous liver parenchyma taken in the vicinity of the tumor (Fig. 1A and B). Approximately, 5 mg of biopsy tissue was placed in advanced DMEM/F-12 (Lot. No. 2522537, GIBCO, Thermo Fisher, Grand Island, NY, USA) with 0.05% fetal bovine serum (FBS) (10082-147, Thermo Fisher Scientific, Stockholm, Sweden) for 3D model organoid generation.

### Organoid culture

Biopsies used for organoid generation were obtained before patients received their first TACE treatment. Tumor and non-tumor liver biopsy samples to be used for the establishment of 3D organoids were approximately 1.6 mm x 5 mm in size, equivalent to a volume of 6.24 mm^3^ (approx. 5 mg) (Fig. 1A-C). They were then cultured with advanced DMEM/F-12 and 0.05% FBS, and directly transferred on ice to the laboratory, before being processed for organoid culture 20 min following retrieval. Mechanical dissociation of the tissue was first carried out, followed by enzymatical digestion with collagenase type IV, to achieve small clusters of cells. Full digestion into individual cells was avoided, as maintaining cell-to-cell interactions intact has been previously shown to promote a more effective derivation^26^. Tumor tissue was mechanically minced and digested briefly (maximum (max) 2–4 min) with Tissue Dissociation Cocktail (2.5 mg/mL collagenase IV (Lot. No. 2357215-1G, GIBCO, Life Technology Corporation, Grand Island, NY, USA), 0.1 mg/mL DNase I solution (Lot. No. 100121466, StemCell Technology, Canada) and Dulbecco’s Modified Eagle’s Medium: Nutrient Mixture F-12 (DMEM/F-12) with 15mM HEPES buffer at 37 °C (Fig. 1A). Purification was then completed through washing and centrifugation. Variations in tumor biopsy size availability for 3D organoid culture prompted differences in procedural performance, depending as well on the content of viable tumor biopsy tissue. Non-tumor organoid culture also slightly differed, as the digestion process and incubation were longer.

Cell clusters were then seeded into Geltrex (Reduced Growth Factor BMM (Basement Membrane Matrix)) (Lot. No. 963702, GIBCO, Grand Island, NY, USA). After gelation of Geltrex, initiation organoid medium was added to the gel dome and has been changed every 2 days for 21 days (Fig. 1D-E). The composition is HepatiCult Organoid Basal Medium (Human) (Lot. No. 1000050870, StemCell Technologies, Canada), Organoid Supplement Solution (Lot. No. 1000078881, StemCell Technologies, Canada), HepatiCult Organoid Growth Supplement (Human) (Lot. No. 1000072496, StemCell Technologies, Canada) and 4-[(1R)-1-aminoethyl]-N-4-pyridinyl-trans-cyclohexanecarboxamide, dihydrochloride (Y-27632) (Lot. No. 1000075285, StemCell Technologies, Canada).

Organoids were passaged by mechanical dissociation to proliferate for 3–5 passages with advanced DMEM/F-12 and 0.04% solution of 25% Bovine Serum Albumin Fraction V, protease free (BSA) (Lot. No. 6995421-50G, Roche Diagnostics Gmbh, Mannheim, Germany) through a 1 ml pipette for 2 min. For each passage, growth organoid medium was added to the gel dome and has been changed every 2 days for 6 days. The composition is HepatiCult Organoid Basal Medium (Human) and HepatiCult Organoid Growth Supplement (Human) (Lot. No. 100–0385, StemCell Technologies, Canada). Cryovials were prepared at regular intervals by dissociating organoids and resuspending in Recovery Cell Culture Freezing Medium (Lot. No. 2450336, GIBCO, Life Technology Corporation, Grand Island, NY, USA) prior to freezing. We prepared frozen stocks of early (≤ P5) passages from all the samples that yielded tumor organoids.

### Drug treatment

Idarubicin hydrochloride (Batch/Lot. No. 9YP5033) was purchased from Pfizer AB, Sollentuna, Sweden, dissolved in Sterile Saline Solution (0.9% Sodium Chloride NaCl) at 500 µM aliquots, and stored at -20 °C. Tumoral and liver organoids were seeded in 48-well plate (Lot. No.178547, Thermo Fisher Scientific, Roskilde, Denmark) at a density of 5 × 10^3^ fragments in 15 µL RGF BME2 droplets. Growth organoid medium was changed every two days to form organoids. PDOs were allowed to grow for 6 days before IDA treatment. Following drug exposure for 24 h, organoid viability was assessed using the CellTiter-Glo^®^ 3D Cell Viability Assay reagent according to the manufacturer’s guidelines (Lot. No. 0000569665, Promega Corporation, Madison, USA). Organoids were exposed to different doses of IDA ranging from 0 to 100 µM. For the comparative study, Doxorubicin Accord 25mL (2 mg/mL; Vnr: 189790, Accord Healthcare Polska Sp.z o.o., Pabianice, Poland). Organoids were exposed to different doses of DOX ranging from 0 to 500 µM. Luminescence was measured on a Synergy H4 Hybrid Reader (BioTek Instruments). Results were normalized to negative control. Curve fitting was performed with Prism (GraphPad) software using nonlinear regression. Pictures were acquired using a Nikon Eclipse TE2000-U microscope equipped with a Nikon D-ECLIPSE camera and Nikon Plan Apo objectives (Plan Fluor 4x/0.130 Ph DL).

### Histological staining

Tumoral and liver organoids were seeded in a 24-well plate (REF. No.83-3922, SARSTEDT, Nümbrecht, Germany) at a density of 1.5 × 10^4^ fragments in 40 µL RGF BME2 droplets. Organoids were collected and pelleted by centrifugation (1200 rpm, 5 min, 4°C) and fixed in 4% paraformaldehyde for 15–30 min. Samples were then washed repeatedly in DPBS (3×, 250 × g, 5 min) to ensure complete removal of fixative prior to storage at 4°C. Following fixation and washing, organoids were pelleted by centrifugation (1200 rpm, 5 min) and encapsulated in liquified HistoGel™ (50–200 µL) by resuspension, allowing the gel to solidify into a compact block prior to paraffin embedding. Encapsulated organoid samples were subsequently transferred into tissue cassettes and subjected to automated paraffin processing to allow paraffin infiltration. Following infiltration, samples were embedded in molten paraffin using an embedding center by orienting the HistoGel™ block in a metal mold, after which the paraffin was allowed to solidify to form a stable block for subsequent sectioning. This procedure was performed according to the protocol elaborated by Bruna Almeida and Luca Urbani from The Roger Williams Institute of Hepatology (London, UK), adapted from Bancroft and Gamble^27^. Paraffin-embedded blocks were then sectioned at a thickness of 5 μm and dried overnight. Prior to staining, sections were deparaffinized and rehydrated. In accordance with standard protocols^28^, Hematoxylin and Eosin (H&E) staining was conducted to assess organoid morphology.

For immunofluorescence staining, antigen retrieval was carried out at 95 °C for 1 h using Diva Decloaker solution (DV2004, Biocare, Gothenburg, Sweden). Sections were then blocked for 30 min and incubated overnight at 4 °C with primary antibodies against epithelial cell adhesion molecule (EpCAM) (AB 71916, Abcam, Cambridge, UK), cytokeratin 19 (CK19) (14-9898-82, ThermoFisher, Stockholm, Sweden), β-catenin (14-2567-82, ThermoFisher, Stockholm, Sweden), platelet endothelial cell adhesion molecule-1 (CD31) (14-0319-82, ThermoFisher, Stockholm, Sweden), and alpha-smooth muscle actin (αSMA) (ab5694, Abcam, Cambridge, UK). Following overnight incubation, sections were incubated for 1 h at room temperature with secondary goat anti-rabbit antibody conjugated to Alexa Fluor™ 488 IgG (H + L) (A-11008, ThermoFisher Scientific, Stockholm, Sweden) and secondary goat anti-mouse antibody Alexa Fluor™ 633 IgG (H + L) (A-21050, ThermoFisher Scientific, Stockholm, Sweden). Nuclei were counterstained with DAPI, and sections were mounted using Fluoromount-G (ThermoFisher Scientific, Stockholm, Sweden). Fluorescent images were acquired using a slide scanner (Carl Zeiss, Axio Scan Z1, Jena, Germany) equipped with a 40×/0.95 Korr M27 Zeiss objective, and representative images were used to characterize organoid structure and marker expression.

### Quantitative RT-PCR of mRNA

Tumoral and liver organoids were seeded in a 24-well plate (REF. No.83-3922, SARSTEDT, Nümbrecht, Germany) at a density of 1.5 × 10^4^ fragments in 40 µL RGF BME2 droplets. RNA was extracted from three domes of PDOs using a RNeasy Universal Mini Kit (74004, Qiagen, Sollentuna, Sweden), following the manufacturer’s manual. More precisely, three technical triplicates were combined in one sample for RNA extraction and qPCR was run on two technical replicates. RNA concentration and purity were determined with a Nanodrop spectrophotometer. The iScript cDNA-synthesis kit (1708891, Bio-Rad, Solna, Sweden) was used for mRNA reverse transcription according to the manufacturer’s guidelines. Primers purchased from ThermoFisher (Supplementary Table 1) were designed with Primer Blast. Amplifications were conducted with Fast SYBR Green (Ref: 4385612, ThermoFisher Scientific) in accordance with the manufacturer’s protocol. Quantification of mRNA levels was performed using QuantStudio 5 (ThermoFisher Scientific) with target gene expression normalized to GAPDH. Using the 2-ΔΔ CT method, fold changes were calculated from average CT values of two technical duplicates for each sample.

### Enzyme-linked immune sorbent assay (ELISA)

Tumoral and liver organoids were seeded in 24-well plate (REF. No.83-3922, SARSTEDT, Nümbrecht, Germany) at a density of 1.5 × 10^4^ fragments in 40 µL RGF BME2 droplets. Conditioned-medium culture from untreated and treated organoids with 2.5 µM IDA for 24 h were used to measure alpha-fetoprotein (AFP) and transforming growth factor-beta 1 (TGF-β1) levels with ELISA kits (EHAFP, ThermoFisher, Stockholm, Sweden) and (88-8350, ThermoFisher, Stockholm, Sweden) respectively according to the manufacturer’s manual. Absorbance was measured at 450 nm with a Synergy H4 Multi-Mode Reader (BioTek Instruments). Results calculations were carried out based on the averages from 3 biological replicates and 2 technical duplicates.

### Live-dead assay

Cell viability in organoids was assessed using the Invitrogen™ LIVE/DEAD™ Viability/Cytotoxicity Kit for mammalian cells (catalogue number L3224, lot 2600120) according to the manufacturer’s instructions. Tumoral and liver organoids were seeded in 24-well plate (REF. No.83-3922, SARSTEDT, Nümbrecht, Germany) at a density of 1.5 × 10^4^ fragments in 40 µL RGF BME2 droplets. Organoids were incubated with a staining solution containing calcein-AM (0.5 µL/mL) and ethidium homodimer-1 (2 µL/mL), prepared in DPBS, for 30 min at 37 °C. Following incubation, organoid domes were rinsed again with DPBS. Images were acquired within 1–2 h after staining using a confocal laser scanning microscope (LSM 700, AxioObserver Carl Zeiss, Jena, Germany) equipped with a Fluor 10×/0.50 M27 objective, and live cells (calcein-AM, turquoise fluorescence) and dead cells (ethidium homodimer-1, violet fluorescence) were identified based on their fluorescence signals. Image analysis was performed using a custom macro to identify, quantify, and calculate the percentage area of dead cells relative to the total live cell area in tumor PDOs following treatment with idarubicin. The macro automatically excluded background signals from the analysis.

### Clinical assessment of tumor necrosis and response

All imaging assessments, tumor response evaluations, and comparative statistical analyses of ex vivo organoids IC_50_ values with corresponding patient clinical data from the TACTida trial were conducted by the Uppsala University Hospital. Methods for the clinical correlations are specified in the next section.

In accordance with the study protocol described by Nyman et al.^25^, imaging assessments were performed before and after TACE. PET/MRI was conducted prior to the first TACE, after the first complete TACE, and after the second TACE in patients with bilobar disease. Contrast-enhanced CT was performed 2–3 weeks after the first TACE to assess lipiodol uptake in the liver. MRI follow-up was conducted after every three completed TACE treatments (every third TACE in unilobar disease and every sixth TACE in bilobar disease). Tumor response was evaluated according to the predefined TACTida protocol using modified Response Evaluation Criteria in Solid Tumors (mRECIST), based on contrast enhancement patterns and changes in viable tumor tissue. MRI parameters included signal intensity, contrast enhancement, and apparent diffusion coefficient (ADC) values. Tumor necrosis was quantified as the percentage change in viable tumor tissue between baseline and post-treatment imaging and was included as an outcome variable describing treatment effect. mRECIST response categories Complete Response (CR), Partial Response (PR), Stable Disease (SD) and Progressive Disease (PD), as well as overall response and disease control, were used to compare tumor response before and after treatment and between patients.

### Correlations with organoid IC_50_ values

IC_50_ values were obtained from PDOs established from tumor and non-tumor tissues. For each patient, IC_50_ values measured in tumor-derived organoids (IC_50_ tumor) were used as the primary ex vivo parameter. IC_50_ values were analyzed both as continuous variables and after stratification into “high” and “low” groups based on the median value. The IC₅₀-derived parameters were evaluated in relation to tumor response, defined by percentage of tumor necrosis and radiological response assessed according to mRECIST criteria, including overall response and disease control rates.

Single-gene correlations were also performed to assess whether dose expression of a specific gene or protein associates with drug sensitivity. Log(IC_50_) values from untreated PDOs were correlated either with individual gene expression from qPCR or with protein concentration from ELISA. qPCR data were normalized using the 2^−ΔΔCt method, and ELISA measurements were used as calculated protein concentrations (pg/ml). For each marker, scatter plots were generated with individual data points and a fitted linear trend line for visualization. Correlation coefficients (ρ) and corresponding p-values were reported. All analyses were conducted using GraphPad Prism.

### Statistical analysis

Data are presented as mean ± standard error of the mean (SEM). Statistical significance was determined using one-way analysis of variance (ANOVA) or an unpaired, two-tailed Student’s T-test, followed by Tukey’s multiple comparison test. p-values < 0.05 were considered statistically significant. IC_50_ values were determined by fitting the curve between the inhibitor and the dose response using the nonlinear regression analysis with the “[inhibitor] vs. response (three parameters)” model in GraphPad Prism 9. Spearman’s rank correlation was conducted to evaluate associations between molecular markers and organoid IC_50_ values. Fisher’s exact test and Spearman correlation were performed to assess associations between clinical parameters and organoid IC_50_ values. Statistical analyses and graphs were made using GraphPad Prism 9. Experiments were done in at least three biological replicates, which we define as parallel measurements of biologically distinct samples taken from independent experiments. Technical replicates were defined as loading the same sample multiple times on the final assay. Outliers were kept in the analyses, unless they were suspected to occur due to technical errors, in which case the experiment was repeated. No formal sample size calculation was performed, as this study was exploratory and based on available PDO material.

## Results

### Generation of HCC patient-derived organoid

Liver and HCC organoids were generated from patient needle biopsies. Out of 65 HCC biopsies, organoids were generated from 42, indicating a general success rate of 64.6%. More specifically, organoids were established from 75.7% (25/33) of collected non-tumor biopsies and 53.1% (17/32) of tumor biopsies. At the patient level, non-tumor and tumor organoids were successfully derived from 71.4% (20/28) and 53.5% (15/28) of patients, respectively. In this cohort, patients were mainly men and underlying chronic liver diseases included HCC etiologies related to excessive alcohol consumption and metabolic dysfunction-associated steatohepatitis (MASH) (Table 1). Three cases of viral hepatitis C infection were also noted. In addition, this cohort includes HCC clinical stages B and C according to the Barcelona Clinic Liver Cancer (BCLC) staging system. The cohort was predominantly Child-Pugh class A (5–6 points; 12/15, 80%), with the remaining patients classified as class B (7–9 points; 3/15, 20%); no patients were class C (10–15 points). Alpha-fetoprotein (AFP) levels were markedly elevated and highly variable, with a mean of 28.40 kIU/L (range 1.1-35.884).

**Table 1:** Baseline characteristics of the patients.

Patient ID	Age	Sex	Cirrhosis*	Cirrhosis etiology*	Child Pugh	BCLC stage	Unilobar (1) Bilobar (2)	Number of tumors	AFP kIE/L
2	77	Male	Yes	HCV	6	B	1	2	26,1
3	78	Male	Yes	Alcohol	6	C	2	12	10,6
5	82	Male	No	-	7	C	1	1	1,1
6	73	Male	Yes	MASH	7	C	2	6	6,6
7	81	Female	No	Alcohol + MASH	5	B	1	1	35,884
10	75	Female	Yes	Cryptogenic	6	C	1	1	49,53
11	71	Female	Yes	Alcohol + MASH	6	C	1	1	3,6
15	72	Male	No	HCV	5	B	1	64	5,8
16	77	Male	Yes	Alcohol + MASH	6	C	2	8	31
21	76	Male	Yes	HCV	5	B	1	2	2,3
22	82	Female	Yes	Alcohol + PBC	7	C	1	1	1,6
23	75	Male	No	MASH	6	B	2	3	18,7
25	85	Male	Yes	PBC	5	B	2	9	11,5
28	74	Male	Yes	Alcohol + Hepatitis C	6	C	2	3	4,1
29	79	Female	Yes	Autoimmune Hepatitis	6	C	1	1	13,73

BCLC, Barcelona Clinic Liver Cancer; MASH, Metabolic Dysfunction-Associated Steatohepatitis; HCV, Hepatitis C virus; PBC, Primary Biliary Cholangitis; AFP, Alpha-fetoprotein.

*Three patients had no clinical signs of cirrhosis but had a history of hepatitis C, excessive alcohol consumption, and/or obesity. These patients were therefore included under the cirrhosis etiology parameter.

### Histological and immunofluorescence characterization reveals partial preservation of architectural and cellular heterogeneity in HCC human patient-derived organoids

**Fig. 2:**
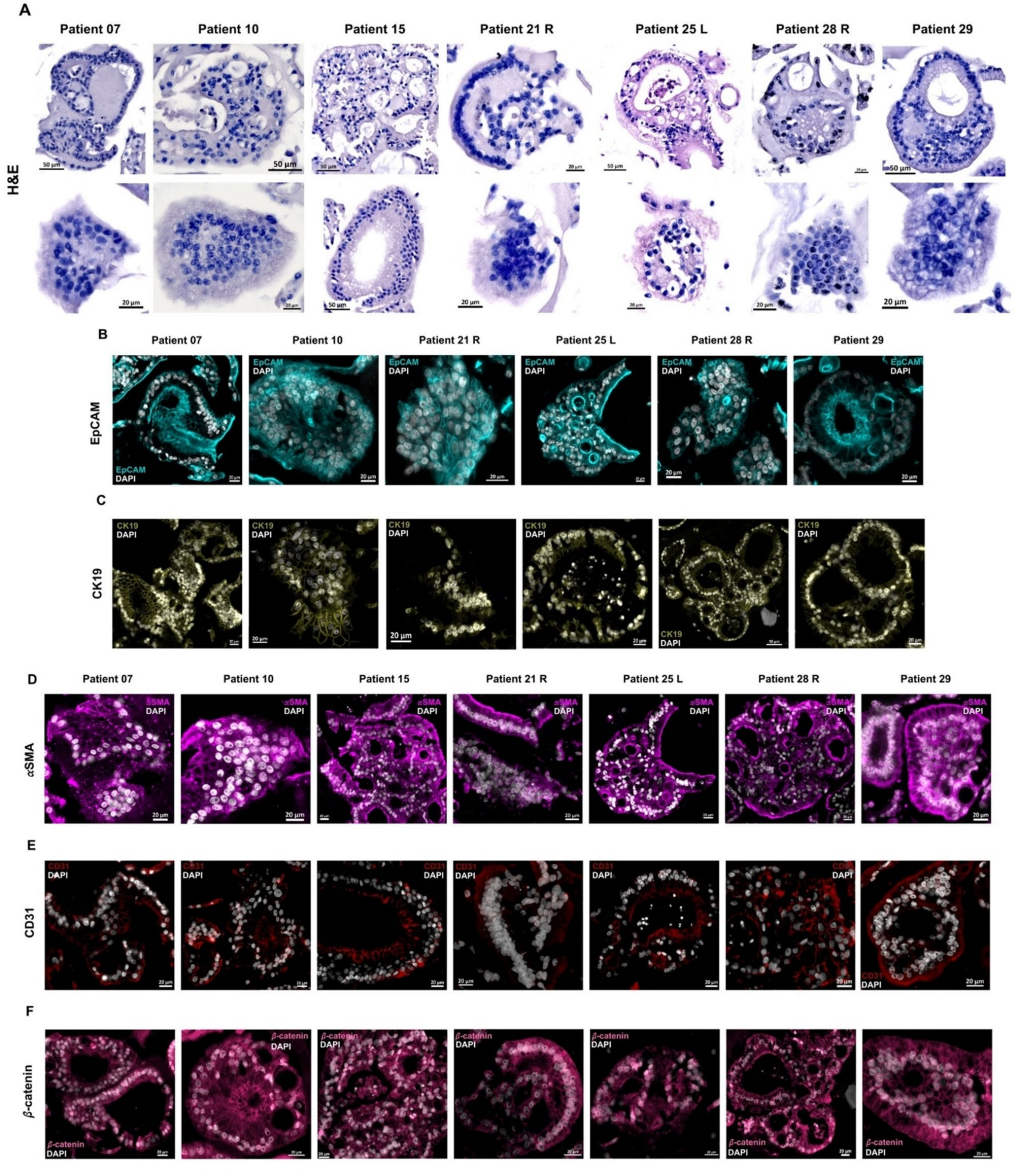
Histological architecture and cellular composition of HCC-derived organoids. **(A)** Representative images of H&E-stained tumor PDOs from seven patients showing trabecular-to-pseudoglandular and solid structures, as well as cytoplasmic and nuclear atypia. Scale bar: 20 μm and 50 μm. Representative immunofluorescence images of EpCAM **(B)**, CK19 **(C)**, αSMA **(D)**, CD31 **(E)**, β-catenin **(F)** stainings in tumor PDOs from seven patients. Scale bar: 20 μm.

H&E staining of PDOs from HCC tumor biopsies revealed marked morphological heterogeneity across cultures (Fig. 2A). Part of the organoids exhibited central lumen-like spaces, representing trabecular-to-pseudoglandular or acinar structures. In contrast, other organoids presented dense, solid growth defined by a compact cell organization without a clear lumen formation or epithelial polarization (Fig. 2A). In addition, a subset of organoids displayed well-defined ductal structures characterized by round lumens lined by a continuous layer of cuboidal cells, indicative of biliary morphology (Supplementary Fig. 1). At the cellular level, organoids were predominantly constituted of polygonal to cuboidal epithelial cells with eosinophilic cytoplasm and enlarged, hyperchromatic nuclei, consistent with malignant epithelial cells with hepatocytic and biliary features (Fig. 2A). These findings underline morphological diversity, similar to what is seen in HCC-patients^29^.

To further define the cellular composition of tumor organoids, several markers were stained by immunofluorescence. Signals of the epithelial markers EpCAM and CK19 were observed across organoid structures, confirming a predominant epithelial cell population with mixed hepatobiliary differentiation characteristics (Fig. 2B-C). Additionally, detection of αSMA and CD31 suggest the presence of non-epithelial cell populations, such as stromal and endothelial cells, within the cultures (Fig. 2D-E). β-catenin expression was also observed, potentially reflecting Wnt signaling activation (Fig. 2F). Overall, histological and immunofluorescence analyses demonstrate that the generated tumor organoids are primarily composed of malignant epithelial cells exhibiting hepatocytic and biliary differentiation, with some retention of cellular constituents associated to the tumor microenvironment (Fig. 2D-E).

### Liver and tumor organoids display variable sensitivity to idarubicin

**Fig. 3:**
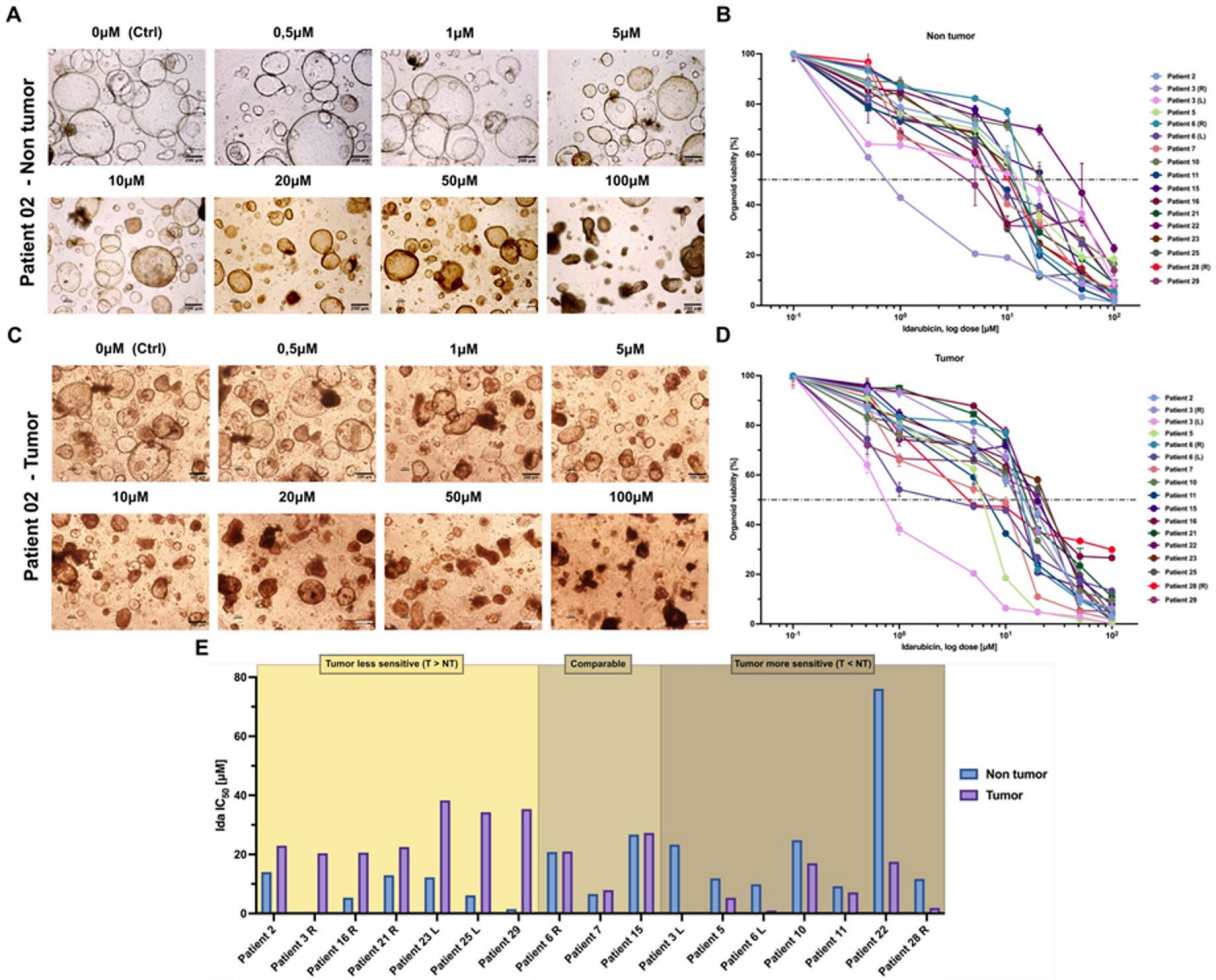
Differential Drug Responses in Patient-Derived Liver and Tumor (HCC) Organoids under Idarubicin Treatment. Non-tumoral and tumoral liver organoids were exposed to IDA at the indicated concentration for 24 h. Representative bright-field images of IDA-treated liver **(A)** and tumor **(C)** organoids (patient 02). Scale bar: 200 μm. Idarubicin reduces cell viability of liver **(B)** and tumor **(D)** organoids in a dose-dependent manner. The dashed line represents the IC_50_. **(E)** IC₅₀ values (µM) of matched tumoral and non-tumoral organoids showed for each patient pair (*n* = 17). Patient pairs are classified according to relative IDA sensitivity as T > NT (tumor less sensitive), comparable IC₅₀ values, or T < NT (tumor more sensitive). Individual dose-response curves and fitted regression lines are shown in Supplementary Fig. 2. Data are presented as the percentage of control and are the mean of 3 biological triplicates and 2 technical duplicates.

To determine drug response in tumoral and non-tumoral liver derived organoid cultures, as well as define the applicability of this experimental approach as a preclinical model, PDOs were treated with a range of IDA concentrations for 24 h (Fig. 3A, C). This exposure period was selected to approximate the early clinically relevant drug exposure window following TACE. Although the exact pharmacokinetic profile of IDA in this setting remains incompletely characterized, pharmacokinetic studies of anthracycline-based TACE have demonstrated sustained local retention during the first 24 h after administration^17,30^. After exposure, organoid viability was assessed using CellTiter-Glo^®^ (Fig. 2B-D, E-F). Ex vivo IDA treatment reduced organoid viability across multiple dose levels in both non-tumorous and tumorous structures (Fig. 3B-D). Nonlinear regression fits showed high R² values across tumor (median R² = 0,9493) and adjacent liver organoids (median R² = 0,9417), supporting confidence in the derived IC₅₀ estimates (Supplementary Table 2a and b; Supplementary Fig. 2). To assess whether IDA exhibited tumor-selective activity, IC₅₀ values were compared in a pairwise manner between tumoral and matched non-tumoral organoids derived from the same patient (Fig. 3E, Supplementary Fig. 2). IC₅₀ values ranged from 0.58 to 38.3 µM across tumor PDOs and from 0.44 to 76.1 µM across non-tumor organoids, indicating marked variability in drug sensitivity. The comparison revealed a heterogeneous response pattern: in 7 of 17 pairs, tumoral organoids showed lower IC₅₀ values than their matched non-tumoral counterparts, consistent with tumor-selective sensitivity to IDA, whereas in 7 others the opposite pattern was observed. The remaining 3 pairs showed comparable IC₅₀ values between tumor and non-tumor organoids, with no clear directional trend (Fig. 3E).

### 2.5µM Idarubicin decreased organoid viability differently across patients

**Fig. 4:**
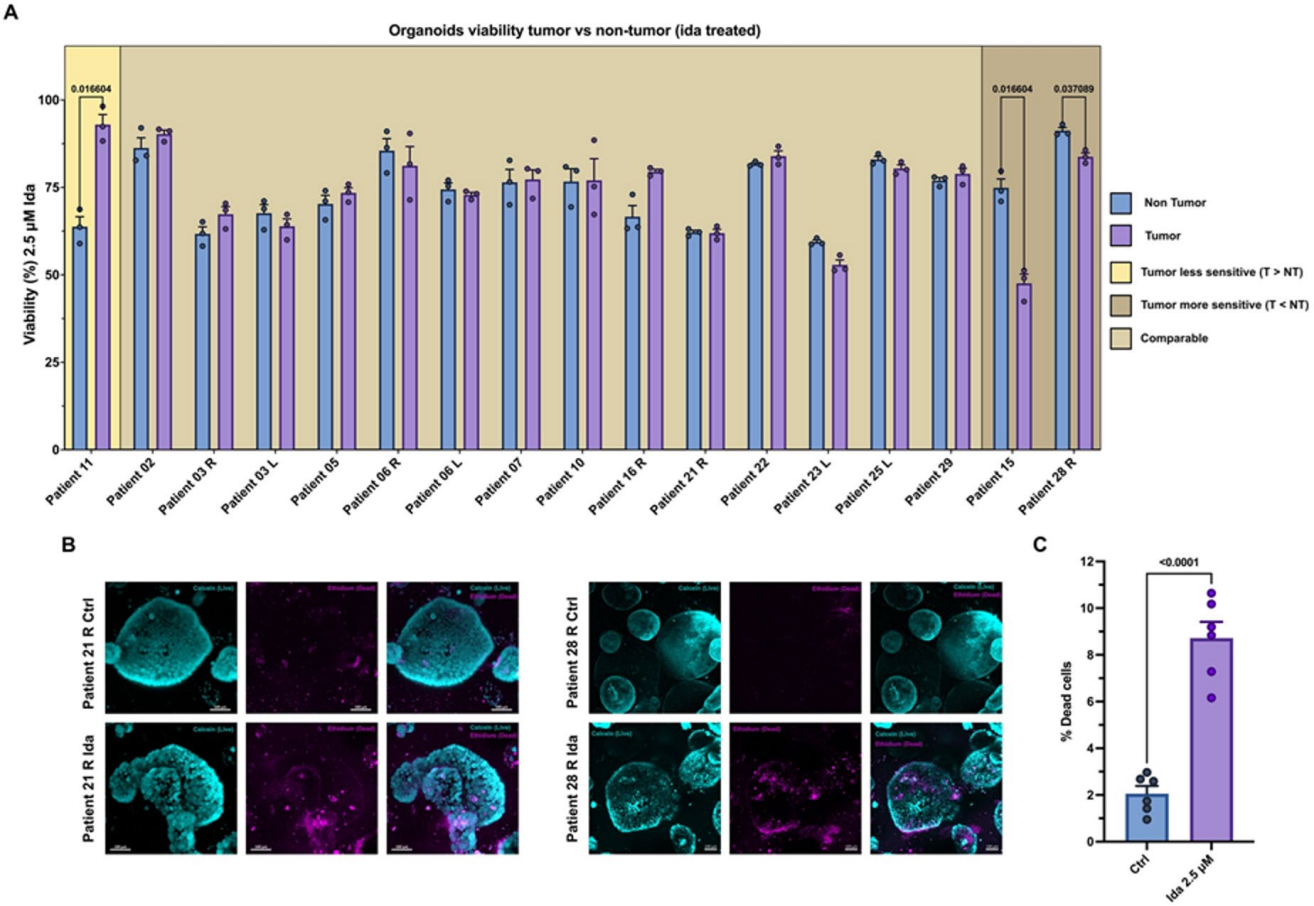
Viability assessment and paired comparison of idarubicin response in patient-derived liver and tumor organoids. (**A**) Paired comparison of IDA response in matched tumor and non-tumor organoids from individual patients. (**B**) Representative images of live/dead assay staining in PDOs following treatment with idarubicin, using Calcein (live, turquoise) and Ethidium Homodimer-1 (dead, violet). Scale bar: 100 μm. (**C**) Quantification of live and dead cells based on fluorescence intensity. Data represent mean ± SEM of 3 biological triplicates and 3 technical duplicates.

Based on dose-finding experiments in our organoid cultures (Supplementary Fig. 2), 2.5 µM idarubicin was selected as a clinically relevant low-micromolar exposure concentration that produced a measurable effect on viability while preserving sufficient organoid integrity for comparative analysis. Patient-derived non-tumor liver and HCC organoids were therefore exposed to 2.5 µM IDA for 24 h to model the local anthracycline exposure that may occur within liver tumors following TACE^31^. Viability was reduced in both non-tumoral and tumoral organoids for all patients, although the magnitude of the response varied substantially between individuals (Supplementary Fig. 3A-D; Supplementary table 3a and 3b). Marked inter-patient heterogeneity in IDA sensitivity was observed, with patients 2, 22, 25, 28R and 29 showing greater resistance to IDA than patients 3R/L, 5, 21 and 23. This pattern was generally consistent across both tumor and non-tumor structures (Supplementary Fig. 3B and 3D; Supplementary table 3a and 3b). Similarly, intra-patient comparison of matched tumor and adjacent liver organoids revealed that tumor PDOs were not uniformly more sensitive to IDA across patients (Fig. 4A). Among patients showing significant differences, tumor organoids exhibited a higher sensitivity to IDA in patients 15 and 28R compared to non-tumor PDOs, whereas patient 3R displayed a higher sensitivity to IDA in non-tumoral organoids than in the tumor PDOs. The remaining patients showed comparable responses between both types (Fig. 4A). To further assess the cytotoxic effect of IDA on HCC PDOs, we also performed a live/dead viability assay following treatment (Fig. 4B). Exposure to 2.5 µM IDA resulted in an increased proportion of dead cells across tumor PDOs compared to untreated controls, consistent with its expected anti-tumoral activity (Fig. 4C).

### Idarubicin exhibits greater potency than doxorubicin in HCC-derived organoids

**Fig. 5:**
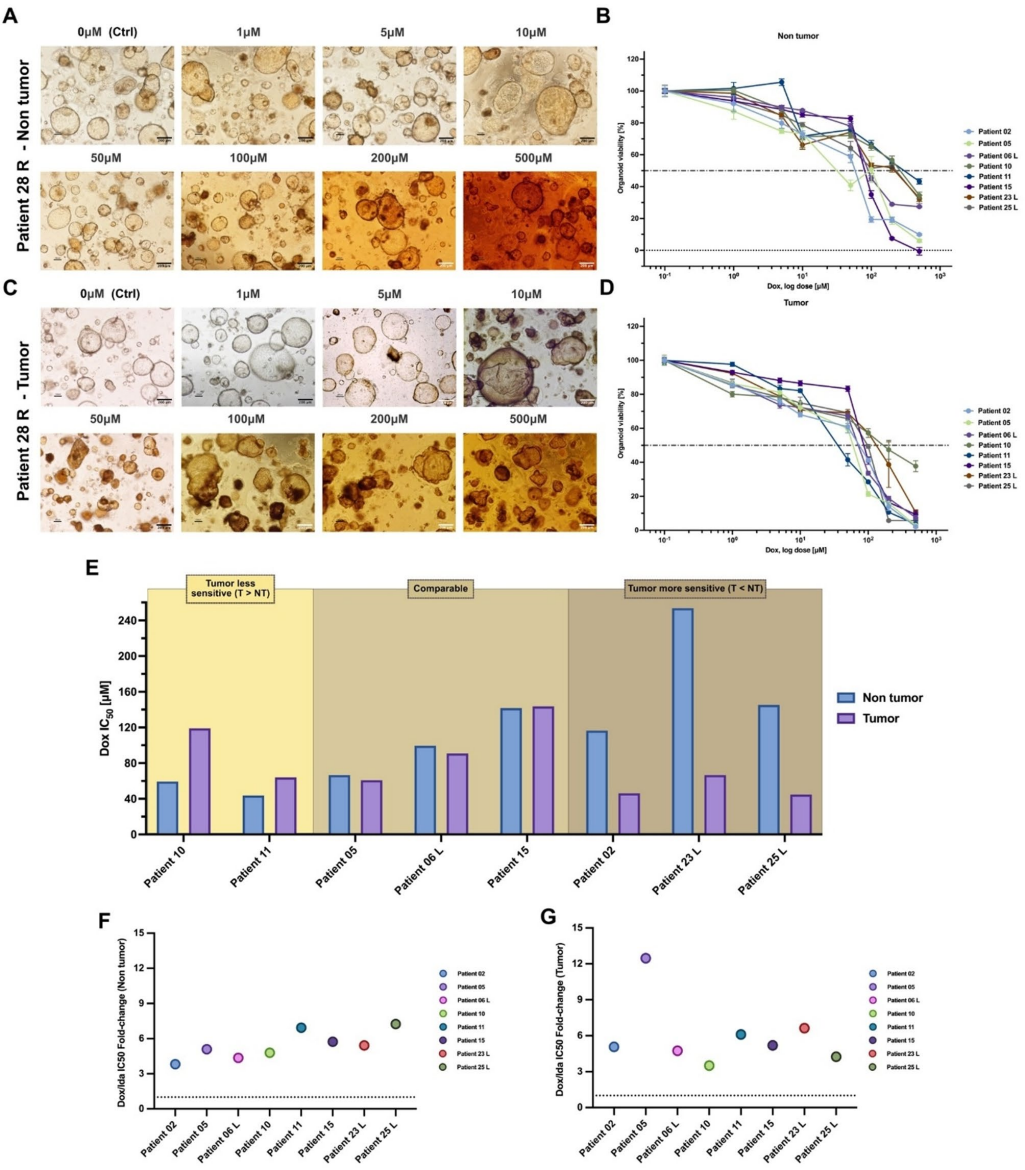
Dose-response analysis of idarubicin (IDA) and doxorubicin (DOX) in HCC-derived organoids. Non-tumoral and tumoral organoids derived from eight HCC patients were treated with increasing concentrations of DOX (0-500 µM) and IDA (0-100 µM) for 24 h. Representative bright-field images of DOX-treated liver **(A)** and tumor **(C)** organoids (patient 28). Scale bar: 200 μm. Dose-response curves of non-tumor **(B)** and tumor **(D)** PDOs treated with DOX. **(E)** IC₅₀ values (µM) of matched tumor and adjacent liver organoids showed for each patient pair (*n* = 17). Patient pairs are classified according to relative DOX sensitivity as T > NT (tumor less sensitive), comparable IC₅₀ values, or T < NT (tumor more sensitive). Individual dose-response curves and fitted regression lines are shown in Supplementary Fig. 3. Fold change in IC₅₀ values between DOX and IDA in non-tumor **(F)** and tumor **(G)** organoids across patients. The dashed line at 1 indicates no change while values above 1 indicate higher DOX relative to IDA. Data represent mean ± SEM of 3 biological triplicates and 2 technical duplicates.

To assess IDA efficacy in relation to standard therapy, we performed a comparative analysis with DOX. Tumor and non-tumor organoids derived from eight HCC patients were exposed to a range of DOX concentrations for 24 h (Fig. 5A, C). DOX treatment resulted in a dose-dependent reduction in organoid viability in both tumor and liver samples (Fig. 5B, D). Nonlinear regression presented mostly high R² values for both tumor and non-tumor structures (Supplementary Table 4a-b; Supplementary Fig. 4). DOX IC₅₀ values ranged from 43,72 to 253,7 µM in tumor PDOs and from 44,82 to 143,6 µM in non-tumor PDOs. Pairwise comparison of matched tumoral and non-tumoral organoids did not reveal a consistent direction in DOX sensitivity. Indeed, tumor organoids were less sensitive than their non-tumor counterparts in 3 of 8 pairs, more sensitive in 2 pairs, and comparable in the remaining 3 pairs (Fig. 5E). When comparing both treatments, IDA demonstrated consistently higher potency, with lower IC₅₀ values observed across all samples (Fig. 5F). Inter-patient variability was observed for both anthracyclines and was of similar magnitude, indicating that although IDA was uniformly more potent, patient-specific differences in drug response were maintained. More specifically, the DOX/IDA IC₅₀ ratio ranged from 4.0 to 12.5, indicating that the IC₅₀ values for DOX were approximately 4- to 12.5-fold higher than those for IDA and support that these human derived PDOs are valuable for ranking drug sensitivities (Fig. 5G) (Supplementary Table 5).

### Idarubicin induces ER stress and inflammatory responses while altering proliferation in tumor PDOs

**Fig. 6:**
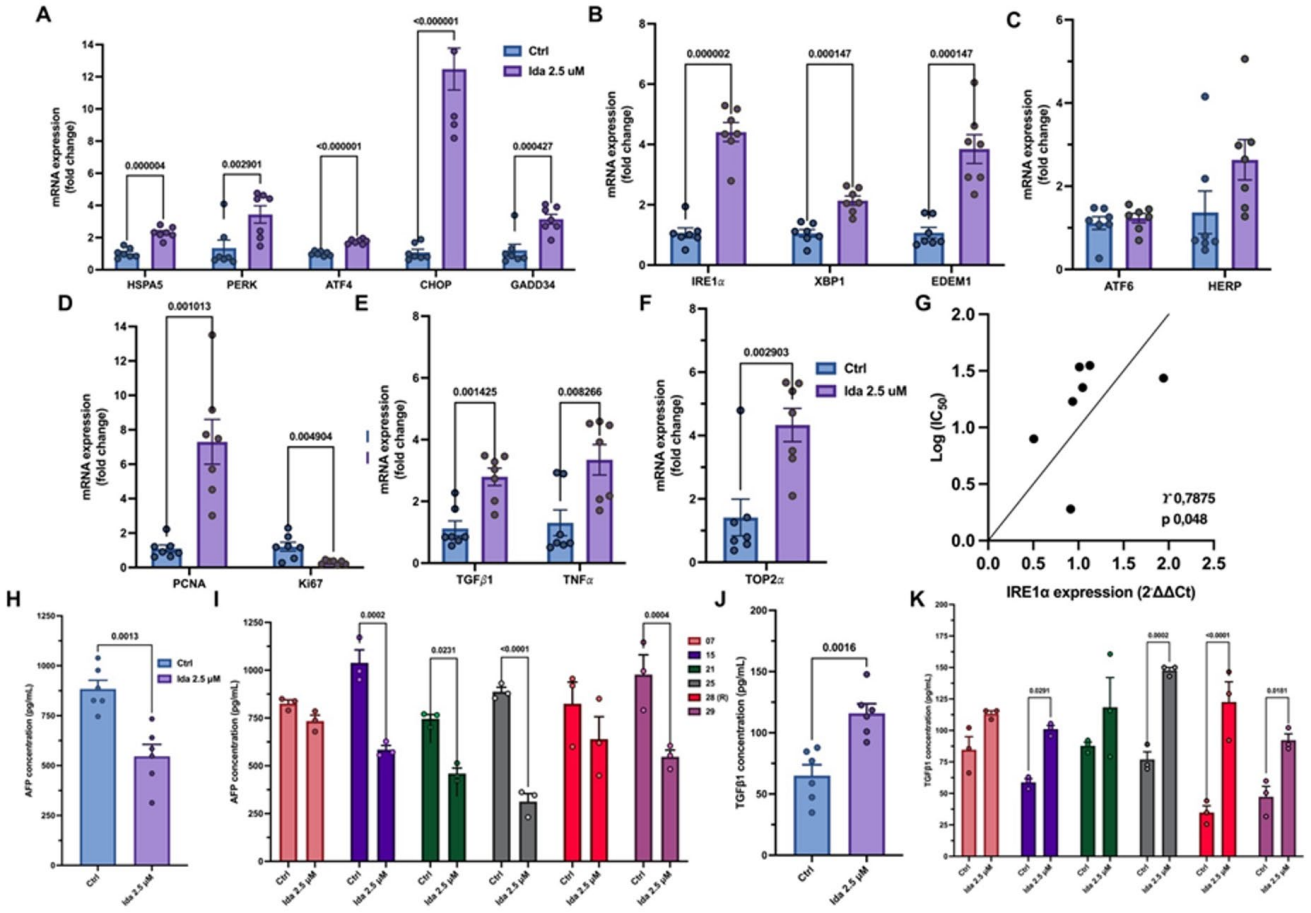
Gene expression and secretory response of HCC-derived organoids following treatment with idarubicin. (**A-C**) qPCR analysis of ER stress-related markers (HSPA5, ATF4, CHOP, GADD34, IRE1α, XBP1, EDEM1, ATF6 and HERP) (**D**) qPCR analysis of proliferation markers (PCNA and Ki67). (**E**) qPCR analysis of inflammatory markers (TGF-β1 and TNF-α). (**F**) qPCR analysis of TOP2α. *N* = 7 patients. (**G**) Scatter plot showing the relationship between IRE1α (ERN1) mRNA expression and IC₅₀ values in untreated HCC PDOs. Correlation was assessed using Spearman’s rank correlation (ρ = 0,7875, p-value = 0,048). Quantification of AFP (**H**) and TGF-β1 (**J**) secretion in tumor PDOs treated with idarubicin. Quantification of AFP (**I**) and TGF-β1 (**K**) secretion per patient in tumor PDOs treated with idarubicin. *N* = 6 patients. Data represent mean ± SEM of 3 biological triplicates and 2 technical duplicates.

To characterize the transcriptional response of PDOs following treatment with IDA, mRNA-expression of selected markers involved in cellular stress, inflammation and proliferation was quantified. As anthracyclines induce oxidative and proteotoxic stress, we first assessed endoplasmic reticulum (ER) stress markers, including components of the PERK (Fig. 6A), IRE1α (Fig. 6B), and ATF6 (Fig. 6C) pathways. Results revealed that IDA exposure increased the expression of HSPA5 (BiP), PERK, ATF4, CHOP, GADD34, IRE1α, XBP1, and EDEM1 (Fig. 6A-B), while ATF6 and HERP were not significantly different between untreated and IDA-treated PDOs (Fig. 6C). Moreover, a significant correlation (*p* = 0.048) was observed in untreated HCC PDOs between baseline IRE1α (ERN1) mRNA expression and IC₅₀ values (Fig. 6G). However, no associations were found with the other ER stress markers (Supplementary Table 6a and 6b). Regarding proliferation, Ki67 expression was significantly decreased (*p* = 0.0049), whereas PCNA levels were markedly increased (Fig. 6D). In parallel, pro-inflammatory markers TGF-β1 and TNF-α showed increased expression levels following exposure to IDA (Fig. 6E). Finally, expression of topoisomerase IIα (TOP2α), the primary molecular target of idarubicin, was assessed and found to be significantly elevated after treatment (*p* = 0.029) (Fig. 6F).

To evaluate whether IDA affected tumor secretory functions, we measured the concentration of alpha fetoprotein (AFP) and TGF-β1 using ELISA (Fig. 6H-K). Findings indicate a considerable decrease in AFP levels across all PDOs following exposure to 2.5 µM IDA, except for patient 7 (Fig. 6H-I). In contrast, TGF-β1 secretion increased after treatment in all PDOs, apart from patient 7, which also showed no significant change (Fig. 6J-K).

### Association of tumor organoid IC_50_ values with clinical response parameters

**Fig. 7:**
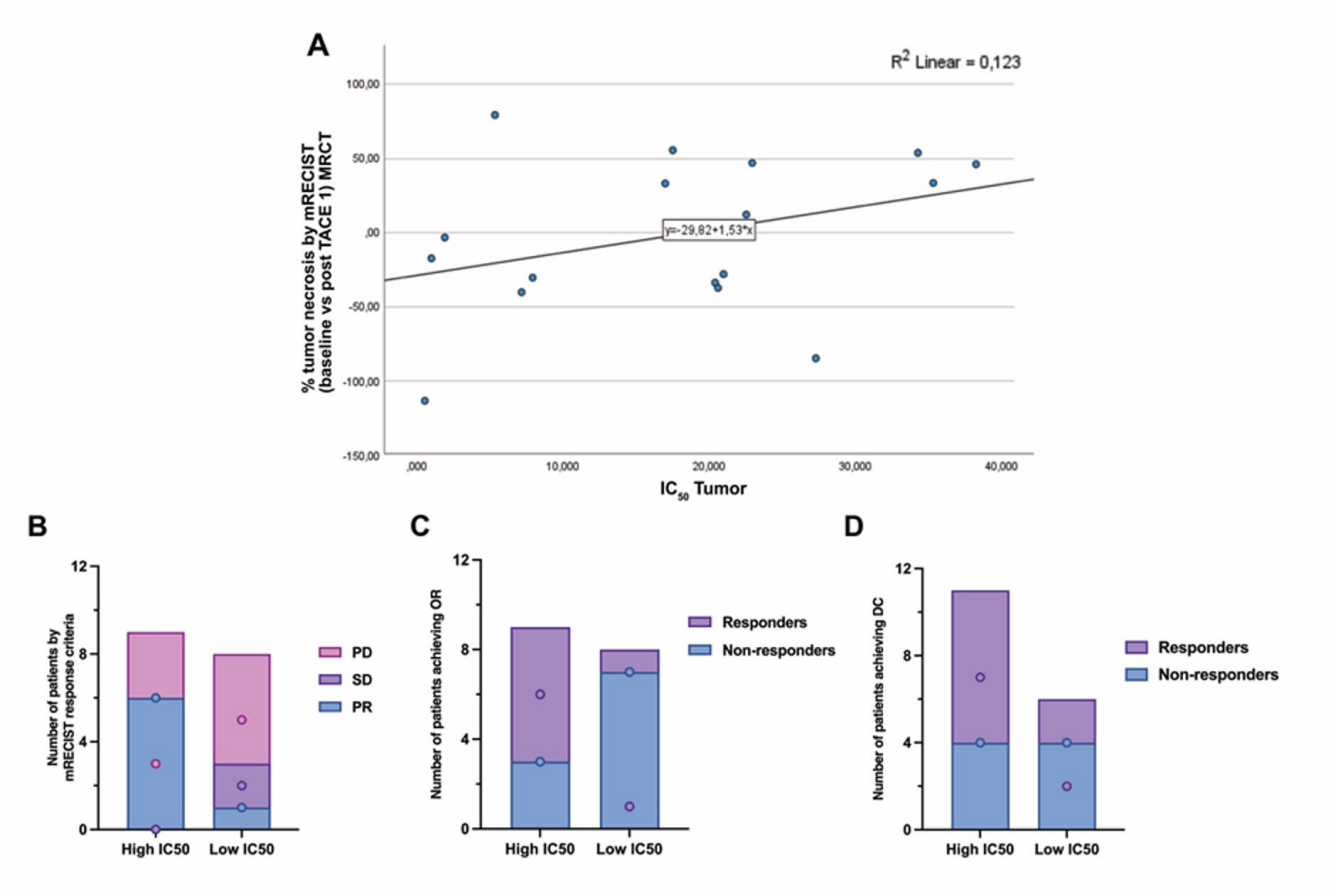
Association between PDO IC₅₀ tumor values and tumor response, defined both as % tumor necrosis and according to mRECIST criteria following TACE in corresponding patients. (**A**) Scatter plot showing the correlation between IC₅₀ tumor values and % tumor necrosis according to mRECIST (baseline vs. post-TACE 1 MR-CT). A linear regression line is shown (y = -29.82 + 1.53x; R² = 0.123. (**B**) Stacked bar chart displaying the number of patients per mRECIST response category Partial Response (PR), Stable Disease (SD) and Progressive Disease (PD), and, stratified by High IC₅₀ vs. Low IC₅₀ tumor groups. (**C**) Stacked bar chart showing the number of OR, categorized as responders and non-responders, stratified by High IC₅₀ vs. Low IC₅₀ groups. (**D**) Stacked bar chart illustrating the number of patients achieving DC, categorized as non-responders and responders stratified by High IC₅₀ vs. Low IC₅₀ groups. Stacked bar charts showing the distribution of clinical response categories within the high- and low-IC₅₀ groups. Bar-segment heights represent cumulative patient counts, whereas open circles indicate the absolute number of patients in each response category. Detailed statistical analyses for all correlation tests are reported in Supplementary Table 7.

We aimed to assess whether ex vivo drug response in tumor PDOs (IC_50_) correlates with clinical outcomes, as quantified by the percentage of tumor necrosis and radiological response according to mRECIST-criteria. No significant correlation was observed between organoid-derived IC_50_ values and tumor necrosis (Fig. 7A). To further explore this relationship, patients were stratified into high (IC_50_ >15) and low (IC_50_ ≤15) groups, based on the median value. Moreover, no association was found between IC_50_ high/low groups and tumor response according to mRECIST, overall response (OR) and disease control (DC) (Fig. 7B and D).

## Discussion

In recent years, 3D organoid models have emerged as a new preclinical experimental system to study tumor biology and therapeutic response, and to various extent preserving tumor architecture and molecular heterogeneity more effectively than established cell lines^19,23,32,33^. In this context, this study aimed to evaluate the reliability of HCC PDOs as a nonclinical experimental ex vivo system, and to investigate the cytotoxic activity and underlying mechanisms of idarubicin (IDA) and its influence on liver derived organoid growth.

Establishing organoids from HCC human patients remains more challenging than for other malignancies, with lower success rates than colorectal (up to 90%)^34^ or pancreatic (75–83%)^35^ cancer PDOs. In our cohort, organoid derivation was achieved in 64.6% of all biopsies (42/65), which is higher than prior HCC organoid studies reporting 33%-38%^19,36^. Our results are comparable to Lo Re et al.^37^(56.7%), while Wong et al.^38^. reported a high short-term culture success rate (94.8%), although the proportion of long-term expandable organoids remained lower (around 30%). These differences in success rate definitions likely result from methodological and biological variability between studies. Moreover, the limited availability of fresh tumor tissue and the scarcity of human biopsies further constrain culture success^19,36^. Strategies to improve derivation efficiency have focused on enhancing culture conditions. Broutier et al.^33^ adjusted the growth factors contained in media composition to limit the outgrowth of normal liver organoids, while Nuciforo et al.^16^ explored the removal and addition of certain compounds to improve HCC proliferation. Nevertheless, these approaches have not fully overcome current limitations as success rates remain variable.

Our findings demonstrate that HCC PDOs partially recapitulate architectural and cellular features of tumors^29^. Histological characterization revealed diverse morphological phenotypes, including lumen-forming, solid, and ductal structures, reflecting HCC heterogeneity. These observations align with findings showing that PDOs mimic patient tumor architecture and histological characteristics^19,33^, including features such as nuclear atypia, eosinophilic cytoplasm, and pseudo-glandular structures^37^. The detection of EpCAM and CK19 further supports the presence of epithelial populations^39^. We also noted hepatocytic and biliary features, with β-catenin signals, suggesting that these organoids maintain a degree of lineage plasticity and Wnt pathway activation. Interestingly, signals for endothelial and stromal markers were also detected, suggesting partial retention of tumor microenvironment components. Although the functional relevance of these populations remains unclear, their potential presence contrasts with studies indicating that organoid systems require stromal co-culture to mimic tumor-stroma interactions^40,41^. Overall, these findings highlight the structural fidelity of HCC PDOs and persistent limitations in fully modelling the tumor microenvironment.

We also investigated the therapeutic response to IDA in comparison to DOX in HCC PDOs. IDA induced a dose-dependent reduction in organoid viability across all samples, with consistently lower IC₅₀ values than DOX in both tumor and non-tumor PDOs. These results align with previous in vitro studies reporting greater cytotoxicity of IDA in HCC and other cancer models, showing lower IC₅₀ values than other anthracyclines and up to a 57-fold higher toxicity index than DOX^15,42^. This has been attributed to IDA’s increased lipophilicity, improved cellular uptake, and enhanced intracellular accumulation^13,43^. In addition, IDA forms a more stable lipiodol emulsion than DOX, supporting its use in TACE settings to improve drug retention in the liver in vivo^17,44^. Collectively, these pharmacological properties of IDA likely contribute to the increased antitumoral activity observed in our PDO model.

However, TACE relies in part on the assumption that the cytotoxic agent itself confers a tumor-specific effect. Yet, our paired PDO analyses showed no consistent intrinsic tumor-selective sensitivity to either IDA or DOX. Responses varied markedly between patients: tumor PDOs were sometimes more sensitive than matched non-tumor PDOs, but in other cases showed similar or lower sensitivity. Although IDA produced lower IC₅₀ values than DOX, this was observed in both tissue types, indicating greater ex vivo cytotoxicity rather than enhanced tumor selectivity. The limited selectivity may partly reflect that the non-tumor PDOs were derived from cirrhotic rather than healthy liver tissue, which could affect the cellular composition of the organoids^45^. It might also reflect the experimental model, as both tumor and non-tumor PDOs are maintained under growth-promoting in vitro conditions and contain actively proliferating cells, which may increase their susceptibility to anthracyclines. Moreover, culture expansion can induce phenotypic drift and reduce mature hepatic functions, potentially altering anthracycline handling^46,47^. Together with the cirrhotic origin of the non-tumor samples, these factors may narrow the apparent ex vivo therapeutic selectivity window.

Translating our ex vivo viability findings into a clinical context remains complex. Although IDA demonstrates greater cytotoxicity in PDOs, this does not necessarily translate into superior therapeutic efficacy. Lerma-Clavero et al.^16^ found no significant difference in tumor burden between IDA and DOX in a chemically induced HCC mouse model, despite using a higher IDA-dose. Clinical studies similarly report comparable response rates between IDA and DOX in TACE^14^, while retrospective data suggest promising response rates and survival outcomes with IDA-based DEB-TACE^48^. These findings highlight that treatment efficacy is determined not only by intrinsic antitumor effects but also by pharmacokinetic and pharmacodynamic parameters, including intratumoral drug concentrations, tissue disposition, exposure duration, and drug retention following locoregional administration, which cannot be fully recapitulated by PDO systems.

Beyond drug response, our study provides insights on the transcriptional changes associated with IDA exposure. We observed transcriptional activation of ER stress pathways with upregulation of main unfolded protein response (UPR) mediators, including PERK, CHOP, and IRE1α, similar to what was found in the study by Lerma-Clavero et al.^16^. The observed increase in mRNA expression of UPR markers aligns with reports demonstrating that anthracyclines induce ER stress through oxidative stress, DNA damage, inflammation, and misfolded proteins accumulation^49,50^. Baseline IRE1α expression correlated with IC₅₀ values in our untreated PDOs, suggesting a role in chemotherapy sensitivity, consistent with studies showing that IRE1α inhibition enhances DOX efficacy^51^. Although these findings provide transcriptional evidence consistent with engagement of ER stress-related responses following IDA exposure, further studies that include protein-level validation and functional analyses will be required to further define the biological effects.

IDA treatment also increased mRNA expression of inflammatory markers TNF-α and the pro-fibrotic marker TGF-β1, suggesting transcriptional engagement of inflammatory and fibrogenic pathways. This is in line with in vivo findings reporting elevated cytokine levels and macrophage recruitment following IDA exposure^16^, as well as our work linking ER-stress-dependent regulation of GP73 to tumor–stroma communication in HCC^52^. Together, these findings suggest that IDA may contribute to remodeling of the hepatic microenvironment by promoting inflammatory and profibrotic signaling.

Changes in proliferation- and DNA damage-associated markers further indicate a complex response to IDA. Our PDOs displayed proliferative activity with increased baseline Ki67 expression, consistent with previous detection via staining in HCC PDOs^37^. Ki67, which marks actively cycling cells, decreased in PDOs after treatment, consistent with reduced proliferation^53^. In contrast, increased PCNA expression may reflect its role in DNA repair rather than enhanced proliferation^54^. The concomitant upregulation of TOP2α may represent a compensatory response to topoisomerase inhibition and the accumulation of TOP2α-DNA cleavage complexes. By preventing DNA religation, IDA induces persistent DNA damage and apoptosis^55,56^. Overall, these changes are more consistent with activation of DNA damage and repair pathways than with increased proliferation, in line with the established mechanism of anthracyclines and the reported association of TOP2α with treatment response and HCC progression^57–59^.

PDO IC₅₀ values did not correlate significantly with tumor necrosis or mRECIST response, consistent with previous liver organoid studies^60^. This could be due to the limited sample size and evaluation following a single TACE procedure may underestimate treatment effects, as multiple interventions (≥ 3) are usually required to achieve significant antitumor activity. Differences between the experimental exposure conditions and the pharmacokinetics of TACE may also have contributed. In the PDO model, drug sensitivity was determined following a spatially uniform 24-hour exposure to defined IDA concentrations. In patients, however, local anthracycline exposure following TACE is dynamic and spatially heterogeneous and is influenced by drug retention and disposition, formation of active metabolite, embolic distribution, vascular anatomy, and the distance between tumor cells and drug-containing vessels or embolic particles. Predictive performance may also be constrained by culture selection: organoids were established from only 45.5% of tumors (15/33), small biopsies may undersample intratumoral heterogeneity, and clonogenic epithelial subpopulations may be preferentially expanded^61,62^. Moreover, TACE exerts its therapeutic effect through both cytotoxic drug delivery and ischemia induced by arterial embolization, mechanisms that are not recapitulated in organoid systems^63^. Indeed, PDOs reflect intrinsic tumor cell drug sensitivity but do not fully capture the complexity of the tumor microenvironment, including stromal, vascular, and immune components, restricting their ability to predict in vivo treatment responses despite demonstrating inter-patient variability in vitro^64^. Therefore, PDO IC₅₀ values may be more informative for comparative drug sensitivity profiling than for direct clinical outcome translation.

To conclude, this study demonstrates that HCC PDOs constitute a biologically relevant ex vivo model that partially preserves structural and phenotypic features of primary tumors and enables comparative drug assessments. Using this system, we rank that IDA exhibits greater cytotoxic potency than DOX in HCC organoids. However, these findings are based on ex vivo sensitivity assays and do not establish clinical superiority, as the relationship between organoid drug response and clinically achievable drug exposure during TACE remains incompletely defined. Mechanistically, our findings indicate that IDA increases mRNA-expression of ER stress markers, providing insight into its mode of action. Future studies combining microenvironmental and in vivo systems are needed to improve the pathophysiological and translational relevance of PDO models.

## Supplementary Information

Below is the link to the electronic supplementary material.

Supplementary Material 1

## Data Availability

The data supporting the findings of this study is available from the corresponding author upon reasonable request. Because of the sensitivity of the data, it is subjected to privacy and ethical restrictions.
