# Supplementary material for: Characterization of hepatocellular carcinoma patient-derived organoids from patients receiving transarterial chemoembolization as a model for preclinical drug response assessment
Source: Sci Rep. 2026 Aug 4;16:24015. doi: 10.1038/s41598-026-65418-3 (PMC13438109; doi:10.1038/s41598-026-65418-3)
Supplement: Supplementary file 1 [file 41598_2026_65418_MOESM1_ESM.docx]

**Supplementary Table 1: Primer list (qPCR)**

| **Gene Name** | **NCBI Reference** | **Species** | **Primer** | **Comments** |
| --- | --- | --- | --- | --- |
| **GAPDH** | NM_001289745.2 | Human | F: GGAGCGAGATCCCTCCAAAAT R: GGCTGTTGTCATACTTCTCATGG | Reference Gene |
| **PERK** | NM_001313915.2 | Human | F: GGAGCAGGGAAGAAAAGGTCA  R: TGGATGACACCAAGGAACCG | ER-stress |
| **CHOP** | NM_001195053.1 | Human | F: GGAACCTGAGGAGAGAGTGTTC  R: GGATAATGGGGAGTGGCTGG | ER-stress |
| **HSPA5** | NM_005347.4 | Human | F: GAAAGAAGGTTACCCATGCAGT  R: CAGGCCATAAGCAATAGCAGC | ER-stress |
| **ATF4** | XM_017028807.2 | Human | F: TGGCCAAGCACTTCAAACCT  R: GTTGTTGGAGGGACTGACCAA | ER-stress |
| **GADD34** | NM_014330.3 | Human | F: GCTGTCACGCAATCCCTTGT  R: CCAGACAGCCAGGAAATGGA | ER-stress |
| **ATF6** | NM_007348.4 | Human | F: TCAGACAGTACCAACGCTTATGC  R: GTTGTACCACAGTAGGCTGAGA | ER-stress |
| **HERP** | NM_001010989.3 | Human | F: CATTTAGACCGAGGCCGGTT  R: AGAGCCTGGCATATCACGTC | ER-stress |
| **IRE1𝛼** | [NM_001433.5](https://www.ncbi.nlm.nih.gov/nucleotide/1519311900?report=gbwithparts) | Human | F: AATTGTGTACCGGGGCATGT  R: TGGAATTGCCGGTCCTTCTC | ER-stress |
| **XBP1** | [NM_001079539.2](https://www.ncbi.nlm.nih.gov/nucleotide/1890335782?report=gbwithparts) | Human | F: TTCTGCTTCTGTCGGGGCA  R: GCTACTCTGTTTTTCAGTTTCCTCC | ER-stress |
| **EDEM1** | XM_011534271.3 | Human | F: CAAGTGTGGGTACGCCACG  R: AAAGAAGCTCTCCATCCGGTC | ER-stress |
| **TGF𝛽1** | NM_000660.7 | Human | F: CACGTGGAGCTGTACCAGAA  R: CGGTAGTGAACCCGTTGATG | Inflammation |
| **TNF𝛼** | [NM_000594.4](https://www.ncbi.nlm.nih.gov/nucleotide/1519314819?report=gbwithparts) | Human | F: GCCCATGTTGTAGCAAACCC  R: TGAGGTACAGGCCCTCTGAT | Inflammation |
| **PCNA** | [NM_002592.2](https://www.ncbi.nlm.nih.gov/nucleotide/33239449?report=gbwithparts) | Human | F: TCTTCCCTTACGCAAGTCTCAG  R: CTTGAGTGCCTCCAACACCT | Proliferation |
| **Ki67** | [XM_054365949.1](https://www.ncbi.nlm.nih.gov/nucleotide/2462519362?report=gbwithparts) | Human | F: AAGAAGCAGAGGGTTGCTCC  R: GAGCGCAGGGATATTCCCTTAT | Proliferation |
| **TOP2𝛼** | [XM_005257632.2](https://www.ncbi.nlm.nih.gov/nucleotide/2217313495?report=gbwithparts) | Human | F: CTGGTGCTAGTAGTGAACCTGT  R: TGTCTGGGCGGAGCAAAATA | DNA Replication |

**Supplementary Figure 1. Histological architecture of HCC-derived organoids**

**(A)** Representative images of H&E‑stained PDOs from seven patients showing ductal and biliary structures. Scale bar: 20 μm and 50 μm.

**Supplementary table 2a: Nonlinear regression parameters for idarubicin dose-response curves in tumoral organoids.**

| **Non-Tumor (IDA)** | **IC_50_** | **95% CI (profile likelihood)** | **R squared** |
| --- | --- | --- | --- |
| **Patient 02** | 14,05 | 9,52 to 20,83 | 0,9075 |
| **Patient 03 R** | 0,44 | 0,37 to 0,50 | 0,9877 |
| **Patient 03 L** | 23,35 | 6,21 to 121,10 | 0,7353 |
| **Patient 05** | 11,93 | 7,62 to 18,57 | 0,9001 |
| **Patient 06 R** | 20,83 | 14,76 to 29,93 | 0,9230 |
| **Patient 06 L** | 9,90 | 6,84 to 14,25 | 0,9439 |
| **Patient 07** | 6,60 | 4,36 to 9,69 | 0,9419 |
| **Patient 10** | 24,83 | 17,66 to 35,61 | 0,9447 |
| **Patient 11** | 9,24 | 7,08 to 11,97 | 0,9662 |
| **Patient 15** | 26,74 | 20,45 to 35,41 | 0,9665 |
| **Patient 16 R** | 5,35 | 3,55 to 7,86 | 0,9388 |
| **Patient 21 R** | 12,98 | 9,03 to 18,66 | 0,9323 |
| **Patient 22** | 76,08 | 27,89 to 486,70 | 0,7890 |
| **Patient 23 L** | 12,27 | 9,70 to 15,51 | 0,9690 |
| **Patient 25 L** | 6,19 | 4,71 to 8,04 | 0,9584 |
| **Patient 28 R** | 11,68 | 8,34 to 16,33 | 0,9417 |
| **Patient 29** | 1,52 | 0,78 to 3,11 | 0,8602 |

| **Tumor (IDA)** | **IC_50_** | **95% CI (profile likelihood)** | **R squared** |
| --- | --- | --- | --- |
| **Patient 02** | 22,95 | 17,26 to 30,88 | 0,9608 |
| **Patient 03 R** | 20,41 | 16,49 to 25,43 | 0,9732 |
| **Patient 03 L** | 0,54 | 0,44 to 0,65 | 0,9805 |
| **Patient 05** | 5,34 | 3,96 to 7,103 | 0,9508 |
| **Patient 06 R** | 20,98 | 14,64 to 30,65 | 0,9175 |
| **Patient 06 L** | 0,99 | 0,470to 4,01 | 0,8533 |
| **Patient 07** | 7,92 | 5,19 to 11,73 | 0,9246 |
| **Patient 10** | 16,99 | 12,07 to 24,11 | 0,9361 |
| **Patient 11** | 7,16 | 5,814to 8,77 | 0,9787 |
| **Patient 15** | 27,30 | 19,59 to 38,81 | 0,9504 |
| **Patient 16 R** | 20,60 | 15,81 to 27,10 | 0,9542 |
| **Patient 21 R** | 22,54 | 16,75 to 30,84 | 0,9493 |
| **Patient 22** | 17,51 | 11,97 to 25,99 | 0,9195 |
| **Patient 23 L** | 38,27 | 27,91 to 53,80 | 0,9610 |
| **Patient 25 L** | 34,29 | 23,10 to 52,76 | 0,9444 |
| **Patient 28 R** | 1,90 | 1,31 to 2,76 | 0,9423 |
| **Patient 29** | 35,34 | 20,79 to 63,75 | 0,8809 |

**Supplementary table 2b: Nonlinear regression parameters for idarubicin dose-response curves in non-tumoral organoids.**

**Supplementary Figure 2. Individual dose-response curves and nonlinear regression fits for idarubicin in matched tumoral and non-tumoral organoids.**

Dose-response curves showing organoid viability (% of untreated control) following 24-hour exposure to a range of IDA concentrations, shown individually for each of the 17 matched tumoral/non-tumoral organoid pairs. Raw data points are shown as symbols; solid lines represent the best-fit nonlinear regression curve used to derive IC₅₀ values shown in Figure 3. Tumor organoids are shown in light blue, non-tumor organoids in navy blue. X-axis: IDA concentration (µM, log dose). Y-axis: viability (%).

**Supplementary Figure 3. 2.5μM idarubicin reduces viability in non-tumoral and tumoral patient-derived organoids with inter-patient variability in drug response.**

Non tumoral and tumoral organoids were exposed to 2.5μM Idarubicin for 24 h. 2.5μM IDA decreased viability in non-tumoral **(A)** and tumoral **(C)** patient organoids. Exposure to 2.5μM idarubicin is different between across liver **(B)** and tumor **(D)** PDOs. Data represent mean ± SEM of 3 biological triplicates and 3 technical duplicates.

**Supplementary table 3a: Summary of statistical tests for non-tumor PDOs treated with idarubicin (*p*-values)**

| Tukey's multiple comparisons test | Mean diff, | 95,00% ci of diff | Summary | Adjusted *P* value |
| --- | --- | --- | --- | --- |
| Patient 02 vs. Patient 03R | 24,54 | 12,14 to 36,94 | **** | <0,0001 |
| Patient 02 vs. Patient 03L | 18,65 | 6,24 to 31,05 | *** | 0,0003 |
| Patient 02 vs. Patient 05 | 15,99 | 3,59 to 28,39 | ** | 0,0029 |
| Patient 02 vs. Patient 06R | 0,78 | -11,62 to 13,18 | ns | >0,9999 |
| Patient 02 vs. Patient 06L | 11,83 | -0,57 to 24,23 | ns | 0,0750 |
| Patient 02 vs. Patient 07 | 9,82 | -2,58 to 22,22 | ns | 0,2590 |
| Patient 02 vs. Patient 10 | 9,59 | -2,80 to 21,99 | ns | 0,2922 |
| Patient 02 vs. Patient 11 | 22,49 | 10,09 to 34,89 | **** | <0,0001 |
| Patient 02 vs. Patient 15 | 11,39 | -1,00 to 23,79 | ns | 0,1005 |
| Patient 02 vs. Patient 16 | 19,64 | 7,23 to 32,04 | *** | 0,0001 |
| Patient 02 vs. Patient 21 | 24,13 | 11,73 to 36,53 | **** | <0,0001 |
| Patient 02 vs. Patient 22 | 4,59 | -7,81 to 16,99 | ns | 0,9911 |
| Patient 02 vs. Patient 23 | 26,85 | 14,45 to 39,26 | **** | <0,0001 |
| Patient 02 vs. Patient 25 | 3,27 | -9,13 to 15,67 | ns | 0,9998 |
| Patient 02 vs. Patient 28R | -4,92 | -17,32 to 7,48 | ns | 0,9827 |
| Patient 02 vs. Patient 29 | 9,33 | -3,07 to 21,73 | ns | 0,3333 |
| Patient 03R vs. Patient 03L | -5,89 | -18,29 to 6,50 | ns | 0,9236 |
| Patient 03R vs. Patient 05 | -8,55 | -20,95 to 3,85 | ns | 0,4737 |
| Patient 03R vs. Patient 06R | -23,76 | -36,17 to -11,36 | **** | <0,0001 |
| Patient 03R vs. Patient 06l | -12,72 | -25,12 to -0,31 | * | 0,0397 |
| Patient 03R vs. Patient 07 | -14,72 | -27,12 to -2,32 | ** | 0,0084 |
| Patient 03R vs. Patient 10 | -14,95 | -27,35 to -2,55 | ** | 0,0070 |
| Patient 03R vs. Patient 11 | -2,05 | -14,45 to 10,35 | ns | >0,9999 |
| Patient 03R vs. Patient 15 | -13,15 | -25,55 to -0,75 | * | 0,0287 |
| Patient 03R vs. Patient 16 | -4,90 | -17,31 to 7,49 | ns | 0,9832 |
| Patient 03R vs. Patient 21 | -0,42 | -12,82 to 11,98 | ns | >0,9999 |
| Patient 03R vs. Patient 22 | -19,96 | -32,36 to -7,55 | **** | <0,0001 |
| Patient 03R vs. Patient 23 | 2,31 | -10,09 to 14,71 | ns | >0,9999 |
| Patient 03R vs. Patient 25 | -21,27 | -33,67 to -8,87 | **** | <0,0001 |
| Patient 03R vs. Patient 28R | -29,46 | -41,86 to -17,06 | **** | <0,0001 |
| Patient 03R vs. Patient 29 | -15,21 | -27,61 to -2,81 | ** | 0,0056 |
| Patient 03L vs. Patient 05 | -2,66 | -15,06 to 9,74 | ns | >0,9999 |
| Patient 03L vs. Patient 06r | -17,87 | -30,27 to -5,47 | *** | 0,0006 |
| Patient 03l vs. Patient 06l | -6,82 | -19,22 to 5,57 | ns | 0,8032 |
| Patient 03L vs. Patient 07 | -8,83 | -21,23 to 3,57 | ns | 0,4212 |
| Patient 03L vs. Patient 10 | -9,06 | -21,46 to 3,34 | ns | 0,3799 |
| Patient 03L vs. Patient 11 | 3,84 | -8,55 to 16,24 | ns | 0,9986 |
| Patient 03L vs. Patient 15 | -7,26 | -19,66 to 5,14 | ns | 0,7276 |
| Patient 03L vs. Patient 16 | 0,99 | -11,41 to 13,39 | ns | >0,9999 |
| Patient 03L vs. Patient 21 | 5,48 | -6,92 to 17,88 | ns | 0,9564 |
| Patient 03L vs. Patient 22 | -14,06 | -26,46 to -1,66 | * | 0,0142 |
| Patient 03L vs. Patient 23 | 8,21 | -4,19 to 20,61 | ns | 0,5417 |
| Patient 03L vs. Patient 25 | -15,38 | -27,78 to -2,97 | ** | 0,0049 |
| Patient 03L vs. Patient 28R | -23,57 | -35,97 to -11,17 | **** | <0,0001 |
| Patient 03L vs. Patient 29 | -9,32 | -21,72 to 3,08 | ns | 0,3355 |
| Patient 05 vs. Patient 06R | -15,21 | -27,61 to -2,81 | ** | 0,0056 |
| Patient 05 vs. Patient 06L | -4,17 | -16,57 to 8,23 | ns | 0,9967 |
| Patient 05 vs. Patient 07 | -6,17 | -18,57 to 6,23 | ns | 0,8944 |
| Patient 05 vs. Patient 10 | -6,40 | -18,80 to 6,00 | ns | 0,8660 |
| Patient 05 vs. Patient 11 | 6,50 | -5,90 to 18,90 | ns | 0,8523 |
| Patient 05 vs. Patient 15 | -4,60 | -17,00 to 7,80 | ns | 0,9908 |
| Patient 05 vs. Patient 16 | 3,65 | -8,75 to 16,05 | ns | 0,9993 |
| Patient 05 vs. Patient 21 | 8,13 | -4,26 to 20,54 | ns | 0,5558 |
| Patient 05 vs. Patient 22 | -11,41 | -23,81 to 0,99 | ns | 0,0996 |
| Patient 05 vs. Patient 23 | 10,86 | -1,53 to 23,26 | ns | 0,1412 |
| Patient 05 vs. Patient 25 | -12,72 | -25,12 to -0,32 | * | 0,0395 |
| Patient 05 vs. Patient 28R | -20,91 | -33,31 to -8,51 | **** | <0,0001 |
| Patient 05 vs. Patient 29 | -6,66 | -19,06 to 5,74 | ns | 0,8288 |
| Patient 06R vs. Patient 06L | 11,05 | -1,35 to 23,45 | ns | 0,1257 |
| Patient 06R vs. Patient 07 | 9,04 | -3,35 to 21,44 | ns | 0,3825 |
| Patient 06R vs. Patient 10 | 8,81 | -3,58 to 21,21 | ns | 0,4239 |
| Patient 06R vs. Patient 11 | 21,71 | 9,31 to 34,11 | **** | <0,0001 |
| Patient 06R vs. Patient 15 | 10,61 | -1,78 to 23,01 | ns | 0,1646 |
| Patient 06R vs. Patient 16 | 18,86 | 6,45 to 31,26 | *** | 0,0003 |
| Patient 06R vs. Patient 21 | 23,35 | 10,95 to 35,75 | **** | <0,0001 |
| Patient 06R vs. Patient 22 | 3,81 | -8,59 to 16,21 | ns | 0,9988 |
| Patient 06R vs. Patient 23 | 26,08 | 13,67 to 38,48 | **** | <0,0001 |
| Patient 06R vs. Patient 25 | 2,49 | -9,91 to 14,89 | ns | >0,9999 |
| Patient 06R vs. Patient 28R | -5,70 | -18,10 to 6,70 | ns | 0,9406 |
| Patient 06R vs. Patient 29 | 8,55 | -3,84 to 20,95 | ns | 0,4734 |
| Patient 06L vs. Patient 07 | -2,01 | -14,41 to 10,40 | ns | >0,9999 |
| Patient 06L vs. Patient 10 | -2,23 | -14,63 to 10,17 | ns | >0,9999 |
| Patient 06L vs. Patient 11 | 10,67 | -1,73 to 23,07 | ns | 0,1594 |
| Patient 06L vs. Patient 15 | -0,43 | -12,84 to 11,97 | ns | >0,9999 |
| Patient 06L vs. Patient 16 | 7,81 | -4,58 to 20,21 | ns | 0,6202 |
| Patient 06L vs. Patient 21 | 12,30 | -0,100 to 24,70 | ns | 0,0537 |
| Patient 06L vs. Patient 22 | -7,24 | -19,64 to 5,16 | ns | 0,7310 |
| Patient 06L vs. Patient 23 | 15,03 | 2,62 to 27,43 | ** | 0,0065 |
| Patient 06L vs. Patient 25 | -8,56 | -20,96 to 3,84 | ns | 0,4725 |
| Patient 06L vs. Patient 28R | -16,74 | -29,15 to -4,34 | ** | 0,0016 |
| Patient 06L vs. Patient 29 | -2,50 | -14,90 to 9,90 | ns | >0,9999 |
| Patient 07 vs. Patient 10 | -0,23 | -12,63 to 12,17 | ns | >0,9999 |
| Patient 07 vs. Patient 11 | 12,67 | 0,27 to 25,07 | * | 0,0411 |
| Patient 07 vs. Patient 15 | 1,57 | -10,83 to 13,97 | ns | >0,9999 |
| Patient 07 vs. Patient 16 | 9,82 | -2,58 to 22,22 | ns | 0,2595 |
| Patient 07 vs. Patient 21 | 14,31 | 1,90 to 26,71 | * | 0,0117 |
| Patient 07 vs. Patient 22 | -5,24 | -17,64 to 7,16 | ns | 0,9701 |
| Patient 07 vs. Patient 23 | 17,03 | 4,63 to 29,43 | ** | 0,0012 |
| Patient 07 vs. Patient 25 | -6,55 | -18,95 to 5,84 | ns | 0,8449 |
| Patient 07 vs. Patient 28R | -14,74 | -27,14 to -2,33 | ** | 0,0083 |
| Patient 07 vs. Patient 29 | -0,49 | -12,89 to 11,91 | ns | >0,9999 |
| Patient 10 vs. Patient 11 | 12,90 | 0,49 to 25,30 | * | 0,0347 |
| Patient 10 vs. Patient 15 | 1,80 | -10,60 to 14,20 | ns | >0,9999 |
| Patient 10 vs. Patient 16 | 10,05 | -2,35 to 22,45 | ns | 0,2289 |
| Patient 10 vs. Patient 21 | 14,53 | 2,13 to 26,94 | ** | 0,0097 |
| Patient 10 vs. Patient 22 | -5,01 | -17,41 to 7,39 | ns | 0,9798 |
| Patient 10 vs. Patient 23 | 17,26 | 4,86 to 29,66 | *** | 0,0010 |
| Patient 10 vs. Patient 25 | -6,32 | -18,72 to 6,07 | ns | 0,8760 |
| Patient 10 vs. Patient 28R | -14,51 | -26,91 to -2,11 | ** | 0,0099 |
| Patient 10 vs. Patient 29 | -0,26 | -12,66 to 12,14 | ns | >0,9999 |
| Patient 11 vs. Patient 15 | -11,10 | -23,50 to 1,30 | ns | 0,1215 |
| Patient 11 vs. Patient 16 | -2,85 | -15,25 to 9,54 | ns | >0,9999 |
| Patient 11 vs. Patient 21 | 1,63 | -10,77 to 14,04 | ns | >0,9999 |
| Patient 11 vs. Patient 22 | -17,91 | -30,31 to -5,50 | *** | 0,0006 |
| Patient 11 vs. Patient 23 | 4,36 | -8,03 to 16,76 | ns | 0,9946 |
| Patient 11 vs. Patient 25 | -19,22 | -31,62 to -6,822 | *** | 0,0002 |
| Patient 11 vs. Patient 28R | -27,41 | -39,81 to -15,01 | **** | <0,0001 |
| Patient 11 vs. Patient 29 | -13,16 | -25,56 to -0,76 | * | 0,0285 |
| Patient 15 vs. Patient 16 | 8,25 | -4,15 to 20,65 | ns | 0,5333 |
| Patient 15 vs. Patient 21 | 12,73 | 0,33 to 25,14 | * | 0,0392 |
| Patient 15 vs. Patient 22 | -6,81 | -19,21 to 5,59 | ns | 0,8062 |
| Patient 15 vs. Patient 23 | 15,46 | 3,06 to 27,86 | ** | 0,0046 |
| Patient 15 vs. Patient 25 | -8,12 | -20,52 to 4,27 | ns | 0,5582 |
| Patient 15 vs. Patient 28R | -16,31 | -28,71 to -3,91 | ** | 0,0022 |
| Patient 15 vs. Patient 29 | -2,06 | -14,46 to 10,34 | ns | >0,9999 |
| Patient 16 vs. Patient 21 | 4,49 | -7,91 to 16,89 | ns | 0,9928 |
| Patient 16 vs. Patient 22 | -15,05 | -27,45 to -2,65 | ** | 0,0064 |
| Patient 16 vs. Patient 23 | 7,22 | -5,18 to 19,62 | ns | 0,7353 |
| Patient 16 vs. Patient 25 | -16,37 | -28,77 to -3,96 | ** | 0,0021 |
| Patient 16 vs. Patient 28R | -24,56 | -36,96 to -12,16 | **** | <0,0001 |
| Patient 16 vs. Patient 29 | -10,31 | -22,71 to 2,09 | ns | 0,1973 |
| Patient 21 vs. Patient 22 | -19,54 | -31,94 to -7,13 | *** | 0,0001 |
| Patient 21 vs. Patient 23 | 2,73 | -9,67 to 15,13 | ns | >0,9999 |
| Patient 21 vs. Patient 25 | -20,86 | -33,26 to -8,45 | **** | <0,0001 |
| Patient 21 vs. Patient 28R | -29,05 | -41,45 to -16,64 | **** | <0,0001 |
| Patient 21 vs. Patient 29 | -14,80 | -27,20 to -2,39 | ** | 0,0079 |
| Patient 22 vs. Patient 23 | 22,27 | 9,86 to 34,67 | **** | <0,0001 |
| Patient 22 vs. Patient 25 | -1,32 | -13,72 to 11,08 | ns | >0,9999 |
| Patient 22 vs. Patient 28R | -9,51 | -21,91 to 2,89 | ns | 0,3055 |
| Patient 22 vs. Patient 29 | 4,75 | -7,65 to 17,15 | ns | 0,9877 |
| Patient 23 vs. Patient 25 | -23,59 | -35,99 to -11,18 | **** | <0,0001 |
| Patient 23 vs. Patient 28R | -31,77 | -44,17 to -19,37 | **** | <0,0001 |
| Patient 23 vs. Patient 29 | -17,52 | -29,92 to -5,12 | *** | 0,0008 |
| Patient 25 vs. Patient 28R | -8,19 | -20,59 to 4,21 | ns | 0,5450 |
| Patient 25 vs. Patient 29 | 6,06 | -6,33 to 18,46 | ns | 0,9066 |
| Patient 28R vs. Patient 29 | 14,25 | 1,84 to 26,65 | * | 0,0122 |

**Supplementary table 3b: Summary of statistical tests for tumoral PDOs treated with idarubicin (*p*-values)**

| Tukey's multiple comparisons test | Mean diff, | 95,00% ci of diff, | Summary | Adjusted *P* value |
| --- | --- | --- | --- | --- |
| Patient 02 vs. Patient 03R | 22,92 | 9,19 to 36,64 | **** | <0,0001 |
| Patient 02 vs. Patient 03L | 26,38 | 12,65 to 40,10 | **** | <0,0001 |
| Patient 02 vs. Patient 05 | 16,83 | 3,10 to 30,56 | ** | 0,0056 |
| Patient 02 vs. Patient 06R | 9,07 | -4,65 to 22,80 | ns | 0,5436 |
| Patient 02 vs. Patient 06L | 17,40 | 3,67 to 31,12 | ** | 0,0037 |
| Patient 02 vs. Patient 07 | 12,98 | -0,74 to 26,71 | ns | 0,0801 |
| Patient 02 vs. Patient 10 | 13,25 | -0,47 to 26,98 | ns | 0,0678 |
| Patient 02 vs. Patient 11 | -2,69 | -16,42 to 11,03 | ns | >0,9999 |
| Patient 02 vs. Patient 15 | 42,70 | 28,97 to 56,42 | **** | <0,0001 |
| Patient 02 vs. Patient 16 | 10,75 | -2,97 to 24,48 | ns | 0,2744 |
| Patient 02 vs. Patient 21 | 28,34 | 14,61 to 42,06 | **** | <0,0001 |
| Patient 02 vs. Patient 22 | 6,32 | -7,40 to 20,05 | ns | 0,9392 |
| Patient 02 vs. Patient 23 | 37,40 | 23,68 to 51,13 | **** | <0,0001 |
| Patient 02 vs. Patient 25 | 9,85 | -3,87 to 23,58 | ns | 0,4073 |
| Patient 02 vs. Patient 28R | 6,49 | -7,23 to 20,22 | ns | 0,9263 |
| Patient 02 vs. Patient 29 | 11,38 | -2,34 to 25,11 | ns | 0,2000 |
| Patient 03R vs. Patient 03L | 3,45 | -10,27 to 17,18 | ns | 0,9999 |
| Patient 03R vs. Patient 05 | -6,08 | -19,82 to 7,63 | ns | 0,9549 |
| Patient 03R vs. Patient 06R | -13,85 | -27,57 to -0,12 | * | 0,0462 |
| Patient 03R vs. Patient 06l | -5,52 | -19,25 to 8,20 | ns | 0,9804 |
| Patient 03R vs. Patient 07 | -9,93 | -23,66 to 3,79 | ns | 0,3941 |
| Patient 03R vs. Patient 10 | -9,66 | -23,39 to 4,05 | ns | 0,4388 |
| Patient 03R vs. Patient 11 | -25,61 | -39,34 to -11,88 | **** | <0,0001 |
| Patient 03R vs. Patient 15 | 19,78 | 6,054 to 33,51 | *** | 0,0006 |
| Patient 03R vs. Patient 16 | -12,17 | -25,89 to 1,55 | ns | 0,1301 |
| Patient 03R vs. Patient 21 | 5,41 | -8,30 to 19,15 | ns | 0,9835 |
| Patient 03R vs. Patient 22 | -16,59 | -30,32 to -2,86 | ** | 0,0067 |
| Patient 03R vs. Patient 23 | 14,49 | 0,75 to 28,21 | * | 0,0302 |
| Patient 03R vs. Patient 25 | -13,06 | -26,79 to 0,66 | ns | 0,0762 |
| Patient 03R vs. Patient 28R | -16,43 | -30,15 to -2,70 | ** | 0,0076 |
| Patient 03R vs. Patient 29 | -11,54 | -25,26 to 2,19 | ns | 0,1846 |
| Patient 03L vs. Patient 05 | -9,54 | -23,27 to 4,17 | ns | 0,4594 |
| Patient 03L vs. Patient 06r | -17,30 | -31,03 to -3,57 | ** | 0,0040 |
| Patient 03l vs. Patient 06l | -8,97 | -22,70 to 4,748 | ns | 0,5604 |
| Patient 03L vs. Patient 07 | -13,39 | -27,12 to 0,332 | ns | 0,0619 |
| Patient 03L vs. Patient 10 | -13,12 | -26,85 to 0,601 | ns | 0,0733 |
| Patient 03L vs. Patient 11 | -29,07 | -42,79 to -15,34 | **** | <0,0001 |
| Patient 03L vs. Patient 15 | 16,32 | 2,59 to 30,05 | ** | 0,0082 |
| Patient 03L vs. Patient 16 | -15,63 | -29,35 to -1,89 | * | 0,0136 |
| Patient 03L vs. Patient 21 | 1,96 | -11,77 to 15,69 | ns | >0,9999 |
| Patient 03L vs. Patient 22 | -20,05 | -33,78 to -6,32 | *** | 0,0005 |
| Patient 03L vs. Patient 23 | 11,03 | -2,69 to 24,75 | ns | 0,2398 |
| Patient 03L vs. Patient 25 | -16,52 | -30,25 to -2,79 | ** | 0,0071 |
| Patient 03L vs. Patient 28R | -19,89 | -33,61 to -6,16 | *** | 0,0005 |
| Patient 03L vs. Patient 29 | -14,99 | -28,72 to -1,26 | * | 0,0213 |
| Patient 05 vs. Patient 06R | -7,75 | -21,48 to 5,96 | ns | 0,7722 |
| Patient 05 vs. Patient 06L | 0,56 | -13,16 to 14,29 | ns | >0,9999 |
| Patient 05 vs. Patient 07 | -3,84 | -17,57 to 9,87 | ns | 0,9996 |
| Patient 05 vs. Patient 10 | -3,57 | -17,30 to 10,15 | ns | 0,9998 |
| Patient 05 vs. Patient 11 | -19,52 | -33,25 to -5,79 | *** | 0,0007 |
| Patient 05 vs. Patient 15 | 25,87 | 12,14 to 39,59 | **** | <0,0001 |
| Patient 05 vs. Patient 16 | -6,07 | -19,80 to 7,64 | ns | 0,9555 |
| Patient 05 vs. Patient 21 | 11,51 | -2,21 to 25,23 | ns | 0,1873 |
| Patient 05 vs. Patient 22 | -10,50 | -24,23 to 3,22 | ns | 0,3081 |
| Patient 05 vs. Patient 23 | 20,57 | 6,84 to 34,30 | *** | 0,0003 |
| Patient 05 vs. Patient 25 | -6,97 | -20,70 to 6,75 | ns | 0,8788 |
| Patient 05 vs. Patient 28R | -10,34 | -24,06 to 3,38 | ns | 0,3318 |
| Patient 05 vs. Patient 29 | -5,44 | -19,17 to 8,28 | ns | 0,9827 |
| Patient 06R vs. Patient 06L | 8,32 | -5,39 to 22,05 | ns | 0,6773 |
| Patient 06R vs. Patient 07 | 3,91 | -9,81 to 17,64 | ns | 0,9995 |
| Patient 06R vs. Patient 10 | 4,18 | -9,54 to 17,91 | ns | 0,9989 |
| Patient 06R vs. Patient 11 | -11,76 | -25,49 to 1,96 | ns | 0,1631 |
| Patient 06R vs. Patient 15 | 33,63 | 19,90 to 47,35 | **** | <0,0001 |
| Patient 06R vs. Patient 16 | 1,68 | -12,05 to 15,41 | ns | >0,9999 |
| Patient 06R vs. Patient 21 | 19,27 | 5,54 to 32,99 | *** | 0,0009 |
| Patient 06R vs. Patient 22 | -2,74 | -16,47 to 10,98 | ns | >0,9999 |
| Patient 06R vs. Patient 23 | 28,33 | 14,61 to 42,06 | **** | <0,0001 |
| Patient 06R vs. Patient 25 | 0,78 | -12,94 to 14,51 | ns | >0,9999 |
| Patient 06R vs. Patient 28R | -2,58 | -16,31 to 11,15 | ns | >0,9999 |
| Patient 06R vs. Patient 29 | 2,31 | -11,41 to 16,04 | ns | >0,9999 |
| Patient 06L vs. Patient 07 | -4,41 | -18,14 to 9,31 | ns | 0,9979 |
| Patient 06L vs. Patient 10 | -4,14 | -17,87 to 9,57 | ns | 0,9990 |
| Patient 06L vs. Patient 11 | -20,09 | -33,82 to -6,36 | *** | 0,0005 |
| Patient 06L vs. Patient 15 | 25,30 | 11,57 to 39,03 | **** | <0,0001 |
| Patient 06L vs. Patient 16 | -6,64 | -20,37 to 7,07 | ns | 0,9126 |
| Patient 06L vs. Patient 21 | 10,94 | -2,78 to 24,67 | ns | 0,2505 |
| Patient 06L vs. Patient 22 | -11,07 | -24,80 to 2,65 | ns | 0,2345 |
| Patient 06L vs. Patient 23 | 20,01 | 6,27 to 33,73 | *** | 0,0005 |
| Patient 06L vs. Patient 25 | -7,54 | -21,27 to 6,18 | ns | 0,8048 |
| Patient 06L vs. Patient 28R | -10,91 | -24,63 to 2,81 | ns | 0,2544 |
| Patient 06L vs. Patient 29 | -6,01 | -19,74 to 7,71 | ns | 0,9591 |
| Patient 07 vs. Patient 10 | 0,26 | -13,46 to 14,00 | ns | >0,9999 |
| Patient 07 vs. Patient 11 | -15,67 | -29,40 to -1,949 | * | 0,0131 |
| Patient 07 vs. Patient 15 | 29,72 | 15,99 to 43,44 | **** | <0,0001 |
| Patient 07 vs. Patient 16 | -2,23 | -15,96 to 11,50 | ns | >0,9999 |
| Patient 07 vs. Patient 21 | 15,36 | 1,62 to 29,08 | * | 0,0165 |
| Patient 07 vs. Patient 22 | -6,65 | -20,38 to 7,07 | ns | 0,9117 |
| Patient 07 vs. Patient 23 | 24,42 | 10,70 to 38,15 | **** | <0,0001 |
| Patient 07 vs. Patient 25 | -3,12 | -16,85 to 10,60 | ns | >0,9999 |
| Patient 07 vs. Patient 28R | -6,49 | -20,22 to 7,23 | ns | 0,9262 |
| Patient 07 vs. Patient 29 | -1,59 | -15,33 to 12,13 | ns | >0,9999 |
| Patient 10 vs. Patient 11 | -15,94 | -29,67 to -2,21 | * | 0,0108 |
| Patient 10 vs. Patient 15 | 29,45 | 15,72 to 43,17 | **** | <0,0001 |
| Patient 10 vs. Patient 16 | -2,50 | -16,23 to 11,23 | ns | >0,9999 |
| Patient 10 vs. Patient 21 | 15,09 | 1,36 to 28,81 | * | 0,0199 |
| Patient 10 vs. Patient 22 | -6,92 | -20,65 to 6,801 | ns | 0,8842 |
| Patient 10 vs. Patient 23 | 24,15 | 10,43 to 37,88 | **** | <0,0001 |
| Patient 10 vs. Patient 25 | -3,39 | -17,12 to 10,33 | ns | >0,9999 |
| Patient 10 vs. Patient 28R | -6,76 | -20,49 to 6,965 | ns | 0,9016 |
| Patient 10 vs. Patient 29 | -1,86 | -15,59 to 11,86 | ns | >0,9999 |
| Patient 11 vs. Patient 15 | 45,39 | 31,66 to 59,12 | **** | <0,0001 |
| Patient 11 vs. Patient 16 | 13,44 | -0,28 to 27,17 | ns | 0,0600 |
| Patient 11 vs. Patient 21 | 31,03 | 17,30 to 44,76 | **** | <0,0001 |
| Patient 11 vs. Patient 22 | 9,01 | -4,70 to 22,74 | ns | 0,5531 |
| Patient 11 vs. Patient 23 | 40,10 | 26,37 to 53,82 | **** | <0,0001 |
| Patient 11 vs. Patient 25 | 12,55 | -1,17 to 26,27 | ns | 0,1042 |
| Patient 11 vs. Patient 28R | 9,18 | -4,54 to 22,91 | ns | 0,5235 |
| Patient 11 vs. Patient 29 | 14,08 | 0,34 to 27,80 | * | 0,0397 |
| Patient 15 vs. Patient 16 | -31,95 | -45,67 to -18,22 | **** | <0,0001 |
| Patient 15 vs. Patient 21 | -14,36 | -28,09 to -0,63 | * | 0,0328 |
| Patient 15 vs. Patient 22 | -36,37 | -50,10 to -22,65 | **** | <0,0001 |
| Patient 15 vs. Patient 23 | -5,29 | -19,02 to 8,43 | ns | 0,9867 |
| Patient 15 vs. Patient 25 | -32,84 | -46,57 to -19,12 | **** | <0,0001 |
| Patient 15 vs. Patient 28R | -36,21 | -49,93 to -22,48 | **** | <0,0001 |
| Patient 15 vs. Patient 29 | -31,32 | -45,04 to -17,59 | **** | <0,0001 |
| Patient 16 vs. Patient 21 | 17,59 | 3,86 to 31,31 | ** | 0,0032 |
| Patient 16 vs. Patient 22 | -4,42 | -18,15 to 9,30 | ns | 0,9979 |
| Patient 16 vs. Patient 23 | 26,65 | 12,93 to 40,38 | **** | <0,0001 |
| Patient 16 vs. Patient 25 | -0,89 | -14,62 to 12,83 | ns | >0,9999 |
| Patient 16 vs. Patient 28R | -4,26 | -17,99 to 9,465 | ns | 0,9986 |
| Patient 16 vs. Patient 29 | 0,63 | -13,09 to 14,36 | ns | >0,9999 |
| Patient 21 vs. Patient 22 | -22,01 | -35,74 to -8,285 | *** | 0,0001 |
| Patient 21 vs. Patient 23 | 9,06 | -4,66 to 22,79 | ns | 0,5445 |
| Patient 21 vs. Patient 25 | -18,48 | -32,21 to -4,75 | ** | 0,0016 |
| Patient 21 vs. Patient 28R | -21,85 | -35,57 to -8,12 | *** | 0,0001 |
| Patient 21 vs. Patient 29 | -16,95 | -30,68 to -3,22 | ** | 0,0051 |
| Patient 22 vs. Patient 23 | 31,08 | 17,35 to 44,80 | **** | <0,0001 |
| Patient 22 vs. Patient 25 | 3,53 | -10,20 to 17,26 | ns | 0,9999 |
| Patient 22 vs. Patient 28R | 0,16 | -13,56 to 13,89 | ns | >0,9999 |
| Patient 22 vs. Patient 29 | 5,05 | -8,66 to 18,78 | ns | 0,9914 |
| Patient 23 vs. Patient 25 | -27,55 | -41,27 to -13,82 | **** | <0,0001 |
| Patient 23 vs. Patient 28R | -30,91 | -44,64 to -17,19 | **** | <0,0001 |
| Patient 23 vs. Patient 29 | -26,02 | -39,75 to -12,29 | **** | <0,0001 |
| Patient 25 vs. Patient 28R | -3,36 | -17,09 to 10,36 | ns | >0,9999 |
| Patient 25 vs. Patient 29 | 1,52 | -12,20 to 15,25 | ns | >0,9999 |
| Patient 28R vs. Patient 29 | 4,89 | -8,83 to 18,62 | ns | 0,9939 |

**Supplementary table 4a: Nonlinear regression parameters for doxorubicin dose-response curves in tumoral organoids.**

| **Non-Tumor (DOX)** | **IC_50_** | **95% CI (profile likelihood)** | **R squared** |
| --- | --- | --- | --- |
| **Patient 02** | 46,11 | 28,64 to 71,65 | 0,9213 |
| **Patient 05** | 60,77 | 25,08 to 131,0 | 0,8507 |
| **Patient 06 L** | 90,84 | 67,11 to 122,5 | 0,9492 |
| **Patient 10** | 119 | 30,13 to 343,4 | 0,7913 |
| **Patient 11** | 64,01 | 21,62 to 159,5 | 0,7999 |
| **Patient 15** | 143,6 | 102,3 to 204,1 | 0,9337 |
| **Patient 23 L** | 66,54 | 8,687 to 178,1 | 0,8088 |
| **Patient 25 L** | 44,82 | 21,25 to 88,97 | 0,8865 |

**Supplementary table 4b: Nonlinear regression parameters for doxorubicin dose-response curves in non-tumoral organoids.**

| **Tumor (DOX)** | **IC_50_** | **95% CI (profile likelihood)** | **R squared** |
| --- | --- | --- | --- |
| **Patient 02** | 116,4 | 77,34 to 174,1 | 0,9322 |
| **Patient 05** | 66,58 | 46,41 to 94,05 | 0,9460 |
| **Patient 06 L** | 99,46 | 64,97 to 150,6 | 0,9222 |
| **Patient 10** | 59,45 | 14,41 to 154,2 | 0,7896 |
| **Patient 11** | 43,72 | 36,16 to 52,79 | 0,9840 |
| **Patient 15** | 141,7 | 100,8 to 201,4 | 0,9336 |
| **Patient 23 L** | 253,7 | 111,1 to 713,3 | 0,8067 |
| **Patient 25 L** | 145,3 | 94,89 to 224,7 | 0,9074 |

**Supplementary Figure 4. Individual dose-response curves and nonlinear regression fits for doxorubicin in matched tumoral and non-tumoral organoids.**

Dose-response curves showing organoid viability (% of untreated control) following 24-hour exposure to a range of DOX concentrations, shown individually for each of the 17 matched tumoral/non-tumoral organoid pairs. Raw data points are shown as symbols; solid lines represent the best-fit nonlinear regression curve used to derive IC₅₀ values shown in Figure 5. Tumor organoids are shown in light blue, non-tumor organoids in navy blue. X-axis: DOX concentration (µM, log dose). Y-axis: viability (%).

**Supplementary table 5: Comparative evaluation of DOX and IDA potency and tumor selectivity in PDOs**

| Patient ID | Tumor IC₅₀ DOX (µM) | Tumor IC₅₀ IDA (µM) | Non-tumor IC₅₀ DOX (µM) | Non-tumor IC₅₀ IDA (µM) | DOX/IDA (Tumor) | DOX/IDA (Non-tumor) |
| --- | --- | --- | --- | --- | --- | --- |
| 2 | 116.40 | 22.95 | 46.11 | 14.05 | 5.07 | 3.28 |
| 5 | 66.58 | 5.34 | 60.77 | 11.93 | 12.47 | 5.09 |
| 6 | 99.46 | 20.98 | 90.84 | 20.83 | 4.74 | 4.36 |
| 10 | 59.45 | 16.99 | 119.00 | 24.83 | 3.50 | 4.79 |
| 11 | 43.72 | 7.16 | 64.01 | 9.24 | 6.11 | 6.93 |
| 15 | 141.70 | 27.30 | 143.60 | 26.74 | 5.19 | 5.37 |
| 23 | 253.70 | 38.27 | 66.54 | 12.27 | 6.63 | 5.42 |
| 25 | 145.30 | 34.29 | 44.82 | 6.19 | 4.24 | 7.24 |

**Supplementary table 6a: Summary of statistical analyses assessing associations between ER stress, inflammation, and proliferation markers and IC₅₀ values in untreated PDOs**

| **Correlation (Ctrl)** | **Spearman r** | **P value** | **P value**  **Summary** | **Significant?**  **(alpha = 0.05)** | **Number of**  **XY pairs** |
| --- | --- | --- | --- | --- | --- |
| **CHOP (qPCR)** | 0 | >0,999 | ns | No | 7 |
| **ATF4 (qPCR)** | 0,4643 | 0,302 | ns | No | 7 |
| **ATF6 (qPCR)** | 0,1786 | 0,713 | ns | No | 7 |
| **EDEM1 (qPCR)** | 0,1429 | 0,783 | ns | No | 7 |
| **HERP (qPCR)** | -0,2143 | 0,662 | ns | No | 7 |
| **PERK (qPCR)** | -0,0714 | 0,906 | ns | No | 7 |
| **IRE1α (qPCR)** | 0,7857 | 0,048 | * | Yes | 7 |
| **HSPA5 (qPCR)** | -0,3929 | 0,396 | ns | No | 7 |
| **XBP1 (qPCR)** | 0,5357 | 0,236 | ns | No | 7 |
| **TGFβ1 (qPCR)** | -0,3929 | 0,396 | ns | No | 7 |
| **TNF𝑎 (qPCR)** | -0,4643 | 0,302 | ns | No | 7 |
| **PCNA (qPCR)** | -0,4286 | 0,354 | ns | No | 7 |
| **Ki67 (qPCR)** | -0,3571 | 0,444 | ns | No | 7 |
| **TOP2𝑎 (qPCR)** | -0,2143 | 0,662 | ns | No | 7 |
| **GADD34 (qPCR)** | -0,4643 | 0,302 | ns | No | 7 |
| **TGF𝛽1 (ELISA)** | -0,0285 | >0,999 | ns | No | 6 |
| **AFP (ELISA)** | 0,6571 | 0,175 | ns | No | 6 |

**Supplementary table 6b: Summary of statistical analyses assessing associations between ER stress, inflammation, and proliferation markers and IC₅₀ values in Treated PDOs (2.5 μM)**

| **Correlation (ida 2.5 μM)** | **Spearman r** | **P value** | **P value Summary** | **Significant?**  **(alpha = 0.05)** | **Number of**  **XY pairs** |
| --- | --- | --- | --- | --- | --- |
| **CHOP (qPCR)** | 0 | >0,999 | ns | No | 7 |
| **ATF4 (qPCR)** | -0,6786 | 0,11 | ns | No | 7 |
| **ATF6 (qPCR)** | -0,5357 | 0,236 | ns | No | 7 |
| **EDEM1 (qPCR)** | -0,4286 | 0,354 | ns | No | 7 |
| **HERP (qPCR)** | -0,3571 | 0,444 | ns | No | 7 |
| **PERK (qPCR)** | -0,3571 | 0,444 | ns | No | 7 |
| **IRE1α (qPCR)** | -0,5357 | 0,236 | ns | No | 7 |
| **HSPA5 (qPCR)** | -0,4643 | 0,302 | ns | No | 7 |
| **XBP1 (qPCR)** | -0,0714 | 0,906 | ns | No | 7 |
| **TGFβ1 (qPCR)** | -0,2143 | 0,662 | ns | No | 7 |
| **TNF𝑎(qPCR)** | -0,3571 | 0,444 | ns | No | 7 |
| **PCNA (qPCR)** | -0,2857 | 0,556 | ns | No | 7 |
| **Ki67 (qPCR)** | -0,3571 | 0,444 | ns | No | 7 |
| **TOP2𝑎 (qPCR)** | 0 | >0,999 | ns | No | 7 |
| **GADD34 (qPCR)** | 0 | >0,999 | ns | No | 7 |
| **TGF𝛽1 (ELISA)** | -0,6571 | 0,175 | ns | No | 6 |
| **AFP (ELISA)** | -0,3714 | 0,497 | ns | No | 6 |

**Supplementary table 7: Summary of values from statistical test for clinical correlations**

| **Predictor** | **Outcome** | **Analysis** | **Test statistic** | **p-value** | **n** |
| --- | --- | --- | --- | --- | --- |
| IC_50_ (tumor) | Tumor necrosis (%) | Spearman correlation | 𝜰 = 0.297 | 0.247 | 17 |
| IC_50_ ratio (tumor / non-tumor) | Tumor necrosis (%) | Spearman correlation | 𝜰 = 0.146 | 0.603 | 15 |
| IC_50_ (tumor, high vs low*) | Tumor necrosis (%) (mRECIST) | Mann-Whitney U | U = 22 | 0.178 | 17 |
| IC_50_ (tumor) | Tumor response (CR), (PR), (SD) & (PD) (mRECIST) | Kruskal-Wallis | H = 2,756 | 0.274 | 17 |
| IC_50_ ratio (tumor / non-tumor) | Tumor response (CR), (PR), (SD) & (PD) (mRECIST) | Kruskal-Wallis | H = 0.504 | 0.796 | 17 |
| IC_50_ (tumor, high vs low*) | Tumor response (CR), (PR), (SD) & (PD) (mRECIST) | Fisher's exact test |  | 0.057 | 17 |
| IC_50_ (tumor) | Tumor response -  Overall Response (OR)**  (mRECIST) | Mann-Whitney U | U = 18 | 0.109 | 17 |
| IC_50_ ratio (tumor / non-tumor) | Tumor response -  Overall Response (OR)**  (mRECIST) | Mann-Whitney U | U = 21 | 0.529 | 17 |
| IC_50_ (tumor, high vs low) | Tumor response -  Overall Response (OR)**  (mRECIST) | Fisher's exact test |  | 0.0498 | 17 |
| IC_50_ (tumor) | Tumor response -  Disease Control (DC)***  (mRECIST) | Mann-Whitney U | U = 22 | 0.200 | 17 |
| IC_50_ (tumor / non-tumor | Tumor response -  Disease Control (DC)***  (mRECIST) | Mann-Whitney U | U = 30,50 | 0.945 | 17 |
| IC_50_ (tumor, high vs low*) | Tumor response -  Disease Control (DC)***  (mRECIST) | Fisher's exact test |  | 0.3348 | 17 |

***IC_50_ High vs low groups** were defined using the median IC_50_ value at 15

****Overall Response (OR):** Responders are defined as patients achieving Complete Response (CR) or Partial Response (PR), while non-responders include those with Stable Disease (SD) or Progressive Disease (PD).

*****Disease Control (DC):** Patients with Complete Remission (CR), Partial Response (PR), or Stable Disease (SD) are considered to have disease control, whereas those with Progressive Disease (PD) are classified as non-responders.

**Supplementary table 8: Source list**

| **Reagent type/resource** | **Designation** | **Source or reference** | **Identifiers** | **Additional information** |
| --- | --- | --- | --- | --- |
| Antibody | αSMA (Rabbit monoclonal) | Abcam | ab5694 | 1:100 |
| Antibody | EpCAM (Rabbit polyclonal) | Abcam | ab71916 | 2 µg/mL |
| Antibody | Cytokeratin 19 (CK19) (Mouse polyclonal) | ThermoFisher | 14-9898-82 | 10 µg/mL |
| Antibody | β-catenin (Mouse Polyclonal) | ThermoFisher | 14-2567-82 | 1 µg/mL |
| Antibody | CD31 (Mouse monocolonal) | ThermoFisher | 14-0319-82 | 2 µg/mL |
| Antibody | Goat anti-rabbit Alexa Fluor 488 | ThermoFisher | A-11008 | 4 µg/ml |
| Antibody | Goat anti-mouse Alexa Fluor 633 | ThermoFisher | A-21050 | 1:1,000 |
| Live-cell stain | LIVE/DEAD™ Viability/Cytotoxicity Kit | Invitrogen™ | L3224 | Calcein-AM (0.5) Ethidium (2 µL/mL) |
| Commercial assay or kit | RNeasy Universal Mini Kit | Qiagen | 74004 |  |
| Commercial assay or kit | iScript cDNA-synthesis | Bio-Rad | 1708891 |  |
| Commercial assay or kit | Fast SYBR Green | ThermoFisher | 4385612 |  |
| Commercial assay or kit | Human alpha-fetoprotein (AFP) ELISA kit | ThermoFisher | EHAFP |  |
| Commercial assay or kit | Human transforming growth factor-beta 1 (TGF-β1) ELISA kit | ThermoFisher | 88-8350 |  |
| Commercial assay or kit | Diva Decloacker solution | Biocare | DV2004 |  |
| Commercial assay or kit | CellTiter-Glo® 3D assay | Promega | G9683 |  |
| Chemical compound, drug | Idarubicin hydrochloride | Pfizer AB | 9YP5033 |  |
| Chemical compound, drug | Doxorubicin Accord | e-apoteket | 28126 |  |
| Chemical compound, drug | Geltrex (Basement Membrane Matrix) | GIBCO | 963702 |  |
| Chemical compound, drug | HepatiCult Organoid Basal Medium (Human) | StemCell Technologies | 1000050870 |  |
| Chemical compound, drug | Organoid Supplement Solution | StemCell Technologies | 1000078881 |  |
| Chemical compound, drug | HepatiCult Organoid Growth Supplement (Human) | StemCell Technologies | 1000072496 |  |
| Chemical compound, drug | 4-[(1R)-1-aminoethyl]-N-4-pyridinyl-trans-cyclohexanecarboxamide, dihydrochloride (Y-27632) | StemCell Technologies | 1000075285 |  |
| Chemical compound, drug | Bovine Serum Albumin Fraction V, protease free (BSA) | Roche Diagnostics | 6995421-50G |  |
| Chemical compound, drug | HepatiCult Organoid Growth Supplement (Human) | StemCell Technologies | 100-0385 |  |
| Chemical compound, drug | Recovery Cell Culture Freezing Medium | GIBCO | 2450336 |  |
